# Anti-Inflammatory Diet Index and risk of renal cell carcinoma

**DOI:** 10.1038/s41416-025-03000-w

**Published:** 2025-04-05

**Authors:** Tahir Taj, Pernilla Sundqvist, Alicja Wolk, Katja Fall, Henrik Ugge

**Affiliations:** 1 Clinical Epidemiology and Biostatistics, School of Medical Sciences, Faculty of Medicine and Health, Örebro University, Örebro, Sweden; 2 Department of Urology, Faculty of Medicine and Health, Örebro University, Örebro, Sweden; 3 Institute of Environmental Medicine, Karolinska Institutet, Stockholm, Sweden

**Keywords:** Risk factors, Renal cell carcinoma

## Abstract

**Introduction:**

A diet rich in fruits, vegetables, coffee, and tea, limited red meat, and moderate alcohol intake may reduce the risk of renal cell carcinoma (RCC). The anti-inflammatory potential of diet has been proposed as a mechanism influencing cancer risk. This study assessed the association between an anti-inflammatory diet and RCC risk.

**Methodology:**

Data from two Swedish cohorts, the Swedish-Mammography-Cohort and the Cohort-of-Swedish-Men, were analysed. Dietary habits were assessed using a 96-item food frequency questionnaire. The Anti-Inflammatory Diet Index (AIDI), composed of 16 food groups (11 anti-inflammatory and 5 pro-inflammatory), was used to score dietary patterns. RCC cases were identified from the Swedish Cancer Register using ICD-10 codes, and Cox proportional hazards models were used to estimate hazard ratios based on AIDI quartiles.

**Results:**

Among 71,421 participants, 431 RCC cases were identified during a 19.7-year follow-up. Higher AIDI scores were associated with a lower RCC risk (HR for Q4 vs. Q1: 0.68, CI: 0.52–0.89). In sex-stratified analyses (p-for heterogeneity = 0.006), the association was stronger in among women (HR: 0.47, CI: 0.30–0.75) but less clear in among men (HR: 0.83, CI: 0.63–1.24).

**Conclusion:**

These data suggest that adherence to an anti-inflammatory diet may confer a reduced risk for RCC, especially among women.

## Introduction

Globally, renal cell carcinoma (RCC) accounts for approximately 90% of all kidney cancer [1], and 2–3% of all malignancies, ranking 6th among men and 10th among women diagnosed with cancer [2]. Suggesting a role of environmental factors, the lifetime risk for developing RCC varies between different parts of the world; the risk is lower in Asia (0.3–0.6%), South America, and Africa (0.2%) compared with Europe (1.3%) and North America (1.8%). [3]. Over the last decades, there has been a significant rise in the incidence of kidney cancers, with over 400,000 new cases and 180,000 annual deaths reported globally [4]. A substantial proportion of these cases came from Europe (99,200 cases and 39,000 deaths) [5, 6]. The accumulated body of evidence indicates that tobacco smoking [3], obesity [7], chronic kidney disease [8], and hypertension [9] are well-established risk factors for RCC. Limited evidence suggests that certain risk factors, such as kidney stones [10], family history of kidney cancer [11], consumption of certain analgesic medications [12, 13], diabetes [14], occupational exposure to asbestos and silica [15], a ketogenic diet (high in fat and low in carbohydrates) and intake of processed meat [16, 17], may be associated with an increased risk of RCC. A diet rich in fruits, fibre-rich vegetables [18, 19], regular coffee and tea consumption [20], physical activity [21], healthy weight [22], and moderate alcohol consumption [23] have, on the other hand, been associated with a reduced risk of RCC. However, the combined evidence regarding the overall influence of diet on RCC risk remains inconclusive [22]. Despite evidence linking diet to RCC, the overall role of diet in RCC remains inconclusive due to several specific gaps in knowledge. First, the existing studies vary greatly in design and focus. For example, one study linking a ketogenic diet to RCC was based on a rat model [8], which limits its direct applicability to humans. Another case-control study explored dietary associations but lacked the robust design of prospective studies, introducing potential recall and selection biases [9].

Furthermore, studies on dietary patterns often focus on specific foods or nutrients rather than overall dietary habits. For instance, one study examining vegetable consumption and RCC included only female participants, reported results on specific vegetable groups rather than total consumption, and was limited by a small follow-up period and few RCC cases [18]. Another study exploring the association between fruit and vegetable intake and RCC yielded inconclusive results, suggesting that while total consumption may not be linked to RCC risk, very low consumption could be associated with higher risk [7].

These limitations highlight gaps in existing research, such as the need for studies with more diverse populations, longer follow-up periods, and a focus on total dietary patterns rather than isolated components. Addressing these gaps could provide a clearer understanding of the role of diet in RCC and help reconcile conflicting findings in the literature.

Chronic inflammation is recognised as a contributing factor in the development of several types of cancer through mechanisms including persistent growth-signalling and oxidative stress [24, 25], and has been proposed as a contributing mechanism in the association between obesity and RCC [7]. It has been theorised that specific nutrients may impact systemic inflammation and, consequently, the risk of RCC, and some studies have suggested a possible association between pro-inflammatory diet and RCC [26–28]. Research into composite measures that offer a more comprehensive evaluation of how an individual’s diet may influence chronic systemic inflammation has yet to be thoroughly investigated concerning the risk of RCC. Recent findings suggest that higher scores on the Empirical Dietary Index for Hyperinsulinemia (EDIH), indicative of diets promoting insulin hypersecretion, are associated with an increased risk of kidney cancer development [HR: 1.12; 95% CI: 1.01, 1.23], while higher scores on the Healthy Eating Index (HEI-2015), reflecting adherence to high-quality diets, are associated with a reduced risk of kidney cancer development [HR: 0.85; 95% CI: 0.77, 0.94] [29]. The evidence linking diet and RCC is limited by a focus on individual nutrients rather than holistic patterns, inconsistencies in dietary indices, and a lack of diverse, longitudinal studies. These gaps highlight the need for more comprehensive research to understand the role of diet in RCC risk.

In this study we aimed to examine the impact of dietary inflammatory potential on RCC risk in a Swedish population-based cohort study, using an empirically-derived dietary anti-inflammatory index.

## Methodology

### Study population

The study utilised data from two cohorts, the Swedish Mammography Cohort (SMC) and the Cohort of Swedish Men (COSM). The SMC was established in 1987 and consisted of all women born between 1914 and 1948 who lived in Västmanlands and Uppsala counties in Sweden’s central healthcare region [30]. The COSM, established in 1997, comprises all men born between 1918 and 1952 who lived in Västmanlands and Örebro counties, also located in the Swedish central healthcare region. Data from the cohorts are available to researchers through the Swedish Infrastructure for Medical Population-Based Life-Course and Environmental Research, SIMPLER (https://www.simpler4health.se). A food frequency questionnaire was administered in 1997 and then in 2009.

All study participants were linked with the Swedish Cancer Register, Swedish Cause of Death Register, and Swedish Population Register using the 12-digit Swedish personal identification number. The date and cause of death were obtained for study participants from the Swedish Cause of Death Register [31]. All deaths in Sweden are registered within 30 days, and a specific cause of death is reported in 96% of the recorded fatalities [31]. Deaths due to RCC were classified according to ICD-10 code C64. Emigration out of Sweden during the study period was retrieved from the Swedish Population Register [32].

From a total population of 83,165 participants, we excluded 11,744 (14%) with either prior cancer before enrolment (*N* = 7192) or due to missing information on covariates (*N* = 4552). In total, the final study population included in the main analyses comprised 71,421 participants, out of whom 35,267 were women and 36,154 were men.

### The Anti-Inflammatory Diet Index (AIDI)

For diet assessment, a 96-item food frequency questionnaire (FFQ), designed to reflect Swedish dietary habits and assess food consumption over the past year, was used at baseline in 1997 and then in 2009. The FFQ enquired study participants regarding how often various food items were consumed on average using eight predefined categories, which range from never to more than three times per day. For every participant, the average energy intake per day was estimated by multiplying the frequency of consumption by the energy content of age-specific portion sizes using composition values from the Swedish Food Administration Database [33]. The development of AIDI has been described in detail earlier [34]. In summary, AIDI categorises various food items based on their anti- or pro-inflammatory potential. AIDI comprises 16 food items or groups, of which 11 are classified as anti-inflammatory and 5 as pro-inflammatory, based on their respective association with the inflammatory marker high-sensitive C-reactive protein (hsCRP) in serum. Foods with anti-inflammatory potential include the following (cut-off value in parenthesis): total fruits and vegetables (≥6 servings/day); tea (≥3 servings/day); coffee (≥2 servings/day); wholegrain bread (≥2 servings/day); breakfast cereal (≥1 serving/day) low-fat cheese (≥1 serving/day); olive and canola oil (>0 servings/day); chocolate (≥1 serving/day); nuts (≥2 servings/week); red wine (2–7 servings/week); and beer (2–14 servings/week). The foods with pro-inflammatory potential were as follows (cut-off value in parenthesis): unprocessed red meat (≤0.5 servings/day); processed red meat (≤0.5 servings/day); offal (no consumption); chips (no consumption); and soft drinks (no consumption). When the cut-off was met for each food category, a score of 1 was allotted, and when not, a score of 0 was given; thus, the AIDI score ranges from 0 to 16. Here, a score of 16 indicates dietary habits with the highest anti-inflammatory potential, and 0 indicates dietary habits with the lowest anti-inflammatory potential. The AIDI scale has been empirically developed using data from a subgroup of the SMC (3503 women, age 56–74 years) [34] and has been used in several Swedish studies [35–37].

### Covariates

In addition to dietary habits, the participants were also enquired about general health conditions such as smoking-habits, height and weight, as well as about comorbidities such as diabetes, hypertension, or hypercholesteremia. Participants were further asked about regular medication use, both as prescribed by health care providers and over-the-counter use of vitamins and dietary supplements. The questionnaire also covered information about education (primary, secondary, or university), employment (full-time or part-time, unemployed, studying, disability pension or retired), and civil status (single, married/cohabiting, divorced, widower). Information regarding chronic kidney disease (CKD) and dialysis was obtained from the National Patient Register; ICD10 code N18 and ICD9 code 585 for CKD, and Swedish classification of healthcare procedures (KVÅ) codes DR016, DR055, DR056 for dialysis. We also utilised a physical activity score, measured in metabolic equivalents (MET) hours per day. The score calculates the reported level of activity of study participants in various areas: at work, at home or during housework, while walking or bicycling, and through exercise, all in the year prior to study enrollment. Additionally, participants were questioned about their inactivity levels, such as time spent watching television or reading, as well as the daily hours spent sleeping, sitting, or lying down. Each type of physical activity received an intensity score in MET hours per day, determined according to the compendium of physical activities. The creation of the score has been thoroughly described previously [38].

### Ascertainment of RCC incidence

RCC cases were identified from the Swedish Cancer Register. Cases with any cancer diagnosis before baseline (except for non-melanoma skin cancer) were excluded from analyses. In Sweden, all newly diagnosed tumours are mandated by law to be reported to the national Swedish Cancer Register according to the National Board of Health and Welfare´s regulations (SOSFS2006:15), and the validity of register data for most common solid tumours is considered high [39]. The Swedish Cancer Register further complies with IARC quality criteria [40]. We identified RCC using the International Classification of Diseases (ICD) 10 codes C64. For analyses using RCC by stage as outcome, we categorised RCC cases into localised (≤T2, N0, M0) or advanced cases (all other), based on TNM-classification from the Swedish Cancer Register [41]. Information on TNM status was only available for diagnoses after 2004. Death due to RCC was identified using the Swedish Cause of Death Register using ICD code C64.

### Statistical analysis

We used Cox proportional hazards models with age as the underlying time scale to examine the associations between AIDI and the risk of RCC. Follow-up started January 1^st^ 1998 and ended at the time of any cancer (except for melanoma skin cancer), date of death, date of emigration, or the end of follow in December 2020, whichever occurred first. We determined potential confounders a priori based on the literature on potential risk factors of RCC and data availability. We adjusted for potential confounders in three steps: Model 1 included age (as the time axis). Model 2 was further adjusted for individual-level covariates, including smoking (pack-years), BMI (as continuous variable), employment status (full-time employed, part-time employed, unemployed, studying, disability pension, retired) and civil status (single, married/cohabiting, divorced, widower), and education level (primary, secondary and university). Model 3 (main model) was further adjusted for such comorbidities as diabetes (yes or no), hypertension (yes or no), hypercholesterolaemia (yes or no), and CKD (yes or no). We used complete case analyses, with the implication that participants with missing exposure or incomplete information on any of the covariates were excluded from all analyses.

Cox proportional hazards regression models were used to estimate the hazard ratios (HRs) and 95% confidence intervals (CIs) for RCC, overall and by stage. AIDI was divided into quartiles with the lowest quartile (most pro-inflammatory diet) as the reference: ≤^5^ (Q1: range 0–5, median 5), 6 (Q2), 7 (Q3), and ≥8 (Q4: range 8–14, median 8). We performed two main analyses: (1) using information from the baseline FFQ in 1997 as the exposure and (2) calculating the cumulative long-term exposure using repeated measurements from 1997 FFQ and the 2009 FFQ, for study participants who answered the 2009 survey. The main analysis included the entire follow-up period (1998–2020). The *p*-value for the trend was calculated using a Cox proportional hazards regression model, with ordinal exposure categories treated as a continuous variable.

In the subsequent secondary analyses, we aimed to discern whether the associations observed could be predominantly attributed to either pro-inflammatory or anti-inflammatory constituents of the AIDI. To achieve this, the primary analytical model was retained; however, the exposure variable was substituted with two modified indices delineating the AIDI into its pro-inflammatory and anti-inflammatory components, respectively. These modified indices were computed utilising the identical thresholds established for the original AIDI, ensuring that elevated scores on either index continued to signify pronounced anti-inflammatory potential. Furthermore, to elucidate the discrete effects of each component, the modified indices were mutually adjusted within the analytical framework. The analysis examined the interaction between Anti-inflammatory Diet Index (AIDI) quartiles and gender to determine if the relationship between AIDI scores and the outcome varied between men and women. The interaction analysis confirmed a significant overall interaction between AIDI scores and sex in relation to RCC risk (*p* = 0.006), indicating that the effect of AIDI differed by sex. Based on the observed interaction, all subsequent analyses were carried out separately for each sex, ensuring that the distinct impact of AIDI on men and women were appropriately accounted for. Due to a large proportion of missing data, self-reported NSAID-use as well as physical activity score, measured in metabolic equivalents (MET) hours per day, were not included in the main analyses. However, we performed sensitivity analyses including these as covariates in the model. We conducted two further sensitivity analyses—restricting the sample to non-smokers and excluding the first two years of follow-up and these largely confirmed the robustness and consistency of the main findings. All statistical analyses were performed using R version 4.1.1 (R Foundation for Statistical Computing, Vienna, Austria) [42].

## Results

During the follow-up, which averaged 19.3 years for women and 18.2 years for men, a total of 431 participants were diagnosed with renal cell carcinoma (RCC). This group comprised 283 men (105 with localised RCC and 84 with advanced RCC) and 148 women (43 with localised RCC and 62 with advanced RCC). In 137 cases (94 men and 43 women) the stage of the disease could not be established, owing to missing information from before 2004.

The mean age at baseline was 61.7 years, with variation across SMC 62.7 years and COSM 60.8 years. The average age at baseline slightly increased with higher AIDI quartiles among females but showed a reverse trend for males, indicating younger participants in higher AIDI quartiles. Significant variations in education levels were observed, with a higher percentage of university-educated individuals in the top quartile (29% for females and 25.2% for males). Smoking pack-years decreased with higher AIDI scores, indicating lesser tobacco use among those with more anti-inflammatory diets. This trend was more pronounced among males. Similarly, BMI categories showed a trend towards lower BMI in higher AIDI quartiles, reflecting healthier body weights. Participants in the highest AIDI quartile, compared to those in the lowest, were more likely to have a university education, engage in walking or cycling for at least 40 minutes per day, use dietary supplements regularly, and were less likely to be current smokers (Table 1).

**Table 1:** Descriptive baseline characteristics of Swedish men and women (stratified) by quartiles of the anti-inflammatory diet index (AIDI; maximum Score=14).

	Female (35267)					Male (36154)				
	Quartiles of AIDI					Quartiles of AIDI				
	3	5	07	13		3	5	07	13	
Characteristics					*p*.over all					*p*.over all
Number of participants	11,448	7515	7049	9255		14,751	8177	6608	6618	
Age at baseline ±SD, years	62.7 (±9.2)	62.9 (±9.3)	63.1 (±9.4)	62.3 (±9.0)	<0.001	59.9 (±9.5)	61.1 (±9.8)	61.6 (±10.0)	61.9 (±9.9)	<0.001
Education					<0.001					<0.001
Primary	5567 (49%)	3326 (44%)	2922 (42%)	2876 (31%)		5539 (37.5%)	2897 (35.4%)	2174 (32.9%)	1681 (25.4%)	
Secondary	4521 (40%)	2967 (40%)	2801 (40%)	3719 (40%)		7328 (49.7%)	4021 (49.2%)	3189 (48.3%)	3272 (49.4%)	
University	1360 (12%)	1222 (16%)	1326 (19%)	2660 (29%)		1884 (12.8%)	1259 (15.4%)	1245 (18.8%)	1665 (25.2%)	
Smoking Pack-years	7.4 (±11.5)	6.6 (±10.7)	6.4 (±10.2)	5.8 (±9.5)	<0.001	13.9 (±16.3)	12.6 (±15.5)	11.1 (±14.8)	9.8 (±13.9)	<0.001
BMI	25.5 (±4.2)	25.1 (±4.0)	24.9 (3.8±)	24.5 (3.6±)		26.2 (±3.6)	25.9 (±3.4)	25.6 (±3.2)	25.2 (±3.0)	
Under weight (<18.5)	194 (1.7%)	125 (%)	107 (1.5%)	158 (1.7%)	<0.001	90 (0.6%)	39 (0.5%)	38 (0.6%)	27 (0.4%)	<0.001
Normal weight (18.5–25)	5606 (49.0%)	4042 (%)	3958 (56.1%)	5582 (60.3%)		5795 (39.3%)	3506 (42.9%)	3075 (46.5%)	3313 (50.1%)	
Over weight (25–30)	4029 (35.2%)	2535 (%)	2334 (33.1%)	2814 (30.4%)		6900 (46.8%)	3823 (46.8%)	2948 (44.6%)	2870 (43.4%)	
Obese (>30)	1619 (11.5%)	813 (%)	650 (9.2%)	701 (7.6%)		1966 (13.3%)	809 (9.9%)	547 (8.3%)	408 (6.2%)	
Employment					<0.001					<0.001
Full time	3075 (26.9%)	2203 (29.3%)	2150 (30.5%)	3274 (35.4%)		7609 (51.6%)	4020 (49.2%)	3219 (48.7%)	3319 (50.2%)	
Part time	1957 (17.1%)	1259 (16.8%)	1104 (15.7%)	1540 (16.6%)		441 (3.0%)	243 (3.0%)	194 (2.9%)	180 (2.7%)	
Unemployed	431 (3.8%)	247 (3.3%)	233 (3.3%)	251 (2.7%)		862 (5.8%)	392 (4.8%)	254 (3.8%)	209 (3.2%)	
Studying	4416 (38.6%)	2962 (39.4%)	2867 (40.7%)	3383 (36.6%)		65 (0.4%)	25 (0.3%)	24 (0.4%)	24 (0.4%)	
Disability pension	1106 (9.7%)	587 (7.8%)	500 (7.1%)	569 (6.2%)		925 (6.3%)	397 (4.8%)	271 (4.1%)	217 (3.3%)	
Retired	463 (4.0%)	257 (3.4%)	195 (2.8%)	238 (2.6%)		4849 (32.9%)	3100 (37.9%)	2646 (40.0%)	2669 (40.3%)	
Ever diagnosed Hypertension					0.473					0.083
Yes	233 (2.0%)	139 (1.9%)	135 (1.9%)	161 (1.7%)		920 (6.2%)	585 (7.2%)	447 (6.8%)	1050 (15.9%)	
Ever diagnosed Hypercholesterolaemia					0.123					0.009
Yes	331 (2.9%)	193 (2.6%)	219 (3.1%)	291 (3.1%)		1433 (9.7%)	851 (10.4%)	633 (9.6%)	730 (11.0%)	
Ever diagnosed with Diabetes					0.004					0.057
Yes	485 (4.2%)	315 (4.2%)	300 (4.3%)	312 (3.4%)		920 (6.2%)	585 (7.2%)	447 (6.8%)	439 (6.6%)	
Energy intake kcal day	1686 (±570)	1711 (±575)	1730 (±557)	1830 (±558)	<0.001	2671 (±928)	2652 (±891)	2657 (±928)	2692 (±840)	0.035
Food consumption, anti-inflammatory items										
Fruits and vegetables^a^	4.1 (±2.3)	4.6 (±2.7)	5.1 (±2.9)	6.4 (±3.3)	0.001	3.3 (±1.9)	3.6 (±2.3)	4.1 (±2.6)	5.0 (±2.9)	0.001
Whole grain bread^a^	3.3 (±3.0)	3.8 (2±2.8)	4.1 (±2.8)	4.3 (3±3.0)	<0.001	4.2 (±3.8)	5.0 (±3.9)	5.1 (±3.7)	5.5 (±3.7)	<0.001
Breakfast cereal^a^	0.6 (±0.6)	0.7 (±0.7)	0.8 (±0.7)	1.0 (±0.7)	0.001	0.5 (±0.6)	0.7 (±0.7)	0.8 (±0.7)	1.1 (±0.8)	0.001
Tea^a^	0.6 (±1.0)	0.6 (±1.2)	0.7 (±1.3)	0.9 (±1.6)	<0.001	0.6 (±1.1)	0.6 (±1.2)	0.7 (±1.4)	0.9 (±1.6)	<0.001
Coffee^a^	2.9 (±2.3)	3.1 (±2.0)	3.2 (±2.3)	3.1 (±1.9)	<0.001	3.3 (±2.5)	3.5 (±2.4)	3.5 (±2.3)	3.4 (±2.0)	<0.001
Chocolate^b^	0.7 (±1.1)	0.9 (±1.4)	0.9 (±1.5)	1.1 (±1.6)	<0.001	0.8 (±1.3)	1.0 (±1.5)	1.1 (±1.7)	1.3 (±1.8)	<0.001
Nuts^b^	0.2 (±05)	0.3 (±0.6)	0.3 (±0.6)	0.4 (±1.1)	<0.001	0.2 (±0.5)	0.3 (±0.7)	0.3 (±1.0)	0.5 (±1.2)	<0.001
Wine^b^	1.0 (±1.6)	1.3 (±1.7)	1.5 (±1.7)	2.1 (±1.9)	0.001	1.0 (±1.6)	1.3 (±1.8)	1.6 (±1.9)	2.2 (±2.1)	0.001
Beer^b^	1.5 (±3.6)	1.9 (±3.5)	2.3 (±3.7)	2.9 (±4.0)	<0.001	5.1 (±7.6)	5.6 (±7.0)	5.7 (±6.4)	6.0 (±5.9)	<0.001
Low fat cheese^b^	0.4 (±1.1)	0.8 (±1.5)	1.0 (±1.9)	1.4 (±1.8)	<0.001	0.3 (±1.2)	0.6 (±1.5)	0.9 (±1.8)	1.3 (±1.9)	<0.001
Use of olive/canola oil^b^	0.1 (±0.3)	0.2 (±0.4)	0.2 (±0.4)	0.4 (±0.5)	0.001	0.1 (±0.2)	0.1 (±0.3)	0.2 (±0.4)	0.3 (±0.5)	0.001
Pro-inflammatory items										
Unprocessed meat^a^	0.5 (±0.4)	0.4 (±0.3)	0.4 (±0.3)	0.4 (±0.3)	<0.001	0.5 (±0.4)	0.5 (±0.4)	0.4 (±0.3)	0.4 (±0.3)	<0.001
Processed meat^a^	0.8 (±0.6)	0.7 (±0.6)	0.6 (±0.6)	0.5 (±0.5)	0.001	0.9 (±0.7)	0.8 (±0.7)	0.7 (±0.7)	0.6 (±0.6)	<0.001
Soft drinks^a^	1.0 (±1.5)	0.8 (±1.5)	0.6 (±1.1)	0.4 (±1.0)	<0.001	1.4 (±1.7)	1.1 (±1.6)	0.9 (±1.5)	0.7 (±1.2)	<0.001
Offal^b^	0.3 (±0.6)	0.2 (±0.6)	0.1 (±0.4)	0.1 (±0.4)	<0.001	0.3 (±0.6)	0.2 (±0.6)	0.1 (±0.5)	0.1 (±0.4)	<0.001
Chips^b^	1.5 (±1.6)	1.3 (±1.7)	1.1 (±1.5)	0.9 (±1.4)	<0.001	1.8 (±1.7)	1.6 (±1.7)	1.5 (±1.9)	1.3 (±1.7)	<0.001

^a^food consumption per day.

^b^ food consumption per week.

Foods with anti-inflammatory potential and their serving definitions include: total fruits and vegetables (≥6 servings/day), tea (≥3 servings/day), coffee (≥2 servings/day), wholegrain bread (≥2 servings/day), breakfast cereal (≥1 serving/day), low-fat cheese (≥1 serving/day), olive and canola oil (>0 servings/day), chocolate (≥1 serving/day), nuts (≥2 servings/week), red wine (2–7 servings/week), and beer (2–14 servings/week). Foods with pro-inflammatory potential and their serving definitions are: unprocessed red meat (≤0.5 servings/day), processed red meat (≤0.5 servings/day), offal (no consumption), chips (no consumption), and soft drinks (no consumption). A score of 1 was allotted when the cut-off for each food category was met, and a score of 0 when it was not, resulting in an AIDI score ranging from 0 to 16.

### AIDI and RCC incidence

Overall, a higher Anti-Inflammatory Diet Index (AIDI) score was associated with a lower risk of renal cell carcinoma (RCC) in a dose-dependent manner (Table 2a). Compared with individuals in the lowest quintile of AIDI (Q1, score 0–5), those in the highest quintile (Q4, score 8–14) had a 31–43% lower risk of RCC, with hazard ratios (HRs) ranging from 0.57 (95% CI: 0.44–0.73) to 0.69 (95% CI: 0.52–0.92) across different models using repeated measures of AIDI (1997 & 2009). The associations were slightly attenuated but remained significant when using baseline AIDI (1997), with HRs between 0.59 (95% CI: 0.45–0.77) and 0.68 (95% CI: 0.52–0.89).

**Table 2:** a: Associations between anti-inflammatory diet index (AIDI) and risk for Renal cell carcinoma (RCC), overall and by stage. HRs (95% CIs) of RCC by quintiles of AIDI in 71,421 Swedish women & Men, 1997–2020. b: Associations between anti-inflammatory diet index (AIDI) and risk for Renal cell carcinoma (RCC), overall and by stage. HRs (95% CIs) of RCC by quintiles of AIDI in 35267, Swedish women, 1997–2020. c: Associations between anti-inflammatory diet index (AIDI) and risk for Renal cell carcinoma (RCC), overall and by stage. HRs (95% CIs) of RCC by quintiles of AIDI in 36154, Swedish men, 1997–2020.

a								
AIDI score	Cases	Person-years	Baseline exposure-1997			Repeated measure of AIDI (1997 & 2009)^¶^		
			Hazard ratios (95% confidence intervals)			Hazard ratios (95% confidence intervals)		
			Model 1^a^	Model 2^b^	Model 3^c^	Model 1^a^	Model 2^b^	Model 3^c^
RCC	431							
Q1 (0–5)	194	485,386	Reference	Reference	Reference	Reference	Reference	Reference
Q2 (6)	95	290,728	0.81 (0.63,1.03)	0.84 (0.66,1.07)	0.85 (0.66,1.08)	0.84 (0.66,1.07)	0.88 (0.69,1.12)	0.88 (0.69,1.13)
Q3 (7)	69	255,865	0.66 (0.50,0.87)	0.71 (0.54,0.94)	0.72 (0.55,0.95)	0.57 (0.44,0.73)	0.61 (0.47,0.79)	0.62 (0.48,0.80)
Q4 (8–14)	73	305,543	0.59 (0.45,0.77)	0.66 (0.51,0.87)	0.68 (0.52,0.89)	0.58 (0.44,0.77)	0.67 (0.51,0.89)	0.69 (0.52,0.92)
*P* value for trend			0.00	0.01	0.02	0.00	0.00	0.00
Localised RCC^d *^	148							
Q1 (0–5)	71	484,505	Reference	Reference	Reference	Reference	Reference	Reference
Q2 (6)	29	290,392	0.68 (0.44,1.05)	0.72 (0.47,1.11)	0.73 (0.47,1.12)	0.71 (0.50,1.15)	0.75 (0.49,1.14)	0.76 (0.50,1.15)
Q3 (7)	21	255,556	0.56 (0.34,0.91)	0.62 (0.38,1.00)	0.62 (0.38,1.02)	0.40 (0.28,0.71)	0.44 (0.27,0.70)	0.44 (0.28,0.71)
Q4 (8–14)	27	305,159	0.59 (0.38,0.93)	0.70 (0.45,1.11)	0.72 (0.46,1.14)	0.64 (0.50,1.26)	0.77 (0.49,1.22)	0.80 (0.50,1.26)
*P* value for trend			0.03	0.14	0.16	0.00	0.01	0.01
Advanced RCC^e *^	146							
Q1 (0–5)	68	484,341	Reference	Reference	Reference	Reference	Reference	Reference
Q2 (6)	26	290,248	0.64 (0.41,1.00)	0.66 (0.42,1.04)	0.66 (0.42,1.04)	0.84 (0.54,1.29)	0.87 (0.56,1.34)	0.87 (0.57,1.35)
Q3 (7)	25	255,556	0.69 (0.44,1.10)	0.74 (0.46,1.17)	0.75 (0.47,1.18)	0.68 (0.44,1.05)	0.73 (0.47,1.12)	0.74 (0.48,1.14)
Q4 (8–14)	27	305,126	0.62 (0.40,0.97)	0.69 (0.44,1.09)	0.70 (0.44,1.11)	0.63 (0.40,1.02)	0.71 (0.43,1.17)	0.73 (0.44,1.20)
*P* value for trend			0.08	0.18	0.21	0.18	0.42	0.47

b								
AIDI score	Cases	Person-years	Baseline exposure-1997			Repeated measure of AIDI (1997 & 2009)^¶^		
			Hazard ratios (95% confidence intervals)			Hazard ratios (95% confidence intervals)		
			Model 1^a^	Model 2^b^	Model 3^c^	Model 1^a^	Model 2^b^	Model 3^c^
RCC	148							
Q1 (0–5)	70	216,628	Reference	Reference	Reference	Reference	Reference	Reference
Q2 (6)	26	143,408	0.56 (0.36, 0.88)	0.59 (0.37, 0.92)	0.59 (0.37, 0.93)	0.75 (0.49, 1.15)	0.78 (0.51, 1.20)	0.79 (0.51, 1.20)
Q3 (7)	27	135,217	0.62 (0.40, 0.96)	0.66 (0.42, 1.03)	0.66 (0.43, 1.03)	0.50 (0.32, 0.78)	0.55 (0.35, 0.85)	0.55 (0.35, 0.85)
Q4 (8–14)	25	183,702	0.42 (0.27, 0.66)	0.47 (0.30, 0.74)	0.47 (0.30, 0.75)	0.49 (0.31, 0.78)	0.56 (0.35, 0.90)	0.56 (0.35, 0.90)
*P* value for trend			0.00	0.01	0.01	0.00	0.02	0.02
Localised RCC^d *^	43							
Q1 (0–5)	21	216,275	Reference	Reference	Reference	Reference	Reference	Reference
Q2 (6)	7	143,274	0.50 (0.21, 1.18)	0.54 (0.23, 1.28)	0.55 (0.23, 1.29)	0.61 (0.27, 1.38)	0.66 (0.29, 1.49)	0.66 (0.29, 1.48)
Q3 (7)	3	135,033	0.23 (0.07, 0.77)	0.25 (0.08, 0.85)	0.26 (0.08, 1.86)	0.30 (0.12, 0.76)	0.34 (0.13, 0.87)	0.34 (0.13, 0.87)
Q4 (8–14)	12	183,622	0.66 (0.32, 1.33)	0.76 (0.36, 1.59)	0.76 (0.36, 1.59)	0.68 (0.32, 1.43)	0.83 (0.37, 1.82)	0.82 (0.37, 1.82)
*P* value for trend			0.06	0.11	0.12	0.08	0.14	0.15
Advanced RCC^e *^	62							
Q1 (0–5)	29	216,314	Reference	Reference	Reference	Reference	Reference	Reference
Q2 (6)	12	143,290	0.63 (0.32, 1.23)	0.65 (0.33, 1.27)	0.65 (0.33, 1.28)	1.04 (0.55, 1.98)	1.07 (0.56, 2.05)	1.07 (0.56, 2.04)
Q3 (7)	15	135,182	0.83 (0.45, 1.55)	0.87 (0.46, 1.63)	0.87 (0.47, 1.64)	0.73 (0.38, 1.40)	0.76 (0.39, 1.48)	0.77 (0.40, 1.51)
Q4 (8–14)	6	183,512	0.24 (0.10, 0.58)	0.26 (0.10, 0.62)	0.26 (0.11, 0.63)	0.39 (0.17, 0.90)	0.42 (0.18, 0.99)	0.43 (0.18, 1.00)
*P* value for trend			0.01	0.02	0.02	0.09	0.15	0.17

c								
AIDI score	Cases	Person-years	Baseline exposure-1997			Repeated measure of AIDI (1997 & 2009)^¶^		
			Hazard ratios (95% confidence intervals)			Hazard ratios (95% confidence intervals)		
			Model 1^a^	Model 2^b^	Model 3^c^	Model 1^a^	Model 2^b^	Model 3^c^
RCC	283							
Q1 (0–5)	124	268,757	Reference	Reference	Reference	Reference	Reference	Reference
Q2 (6)	69	147,320	0.99 (0.74, 1.33)	1.01 (0.75, 1.36)	1.02 (0.76, 1.37)	0.91 (0.68, 1.23)	0.93 (0.69, 1.26)	0.95 (0.70, 1.28)
Q3 (7)	42	120,648	0.72 (0.51, 1.03)	0.76 (0.53, 1.08)	0.77 (0.54, 1.09)	0.63 (0.46, 0.86)	0.66 (0.48, 0.91)	0.66 (0.48, 0.92)
Q4 (8–14)	48	121,841	0.81 (0.58, 1.13)	0.87 (0.62, 1.22)	0.83 (0.63, 1.24)	0.74 (0.52, 1.48)	0.80 (0.56, 1.15)	0.82 (0.57, 1.18)
*P* value for trend			0.22	0.40	0.43	0.02	0.07	0.08
Localised RCC^d *^	105							
Q1 (0–5)	50	268,230	Reference	Reference	Reference	Reference	Reference	Reference
Q2 (6)	22	147,118	0.80 (0.48, 1.32)	0.82 (0.50, 1.36)	0.83 (0.50, 1.37)	0.78 (0.48, 1.28)	0.80 (0.49, 1.31)	0.82 (0.50, 1.34)
Q3 (7)	18	120,524	0.79 (0.46, 1.36)	0.84 (0.49, 1.44)	0.84 (0.49, 1.44)	0.48 (0.28, 0.82)	0.50 (0.29, 0.86)	0.50 (0.29, 0.87)
Q4 (8–14)	15	121,537	0.65 (0.36, 1.15)	0.72 (0.40, 1.30)	0.72 (0.40, 1.30)	0.73 (0.42, 1.27)	0.82 (0.47, 1.45)	0.84 (0.48, 1.49)
*P* value for trend			0.46	0.68	0.69	0.06	0.10	0.11
Advanced RCC ^e *^	84							
Q1 (0–5)	39	26,807	Reference	Reference	Reference	Reference	Reference	Reference
Q2 (6)	14	146,958	0.65 (0.35, 1.19)	0.67 (0.36, 1.23)	0.67 (0.36, 1.24)	0.70 (0.39, 1.26)	0.72 (0.40, 1.31)	0.73 (0.40, 1.32)
Q3 (7)	10	120,374	0.56 (0.28, 1.12)	0.60 (0.30, 1.20)	0.60 (0.30, 1.21)	0.65 (0.37, 1.15)	0.69 (0.39, 1.24)	0.70 (0.39, 1.25)
Q4 (8-14)	21	121,614	1.15 (0.68, 1.95)	1.26 (0.73, 2.18)	1.28 (0.74, 2.21)	0.93 (0.52, 1.69)	1.04 (0.57, 1.93)	1.07 (0.58, 1.97)
P value for trend			0.14	0.13	0.13	0.40	0.43	0.43

^a^Model1 adjusting for age.

^b^Model2 adjusting for age, smoking (pack years), BMI, education (primary, secondary, university), employment status (full time, part time, unemployed, studying, disability pension, retired) and average caloric intake (centred at the mean by sex).

^c^Model3 adjusting for age, smoking (pack years), BMI, education (primary, secondary, university), employment status (full time, part time, unemployed, studying, disability pension, retired), diabetes, hypertension, hypercholesterolaemia, chronic kidney disease status (Yes, No), and average caloric intake (centred at the mean by sex).

^d^Total RCC excluding advanced RCC (T3 = < or N1 or M1).

^e^advanced RCC (T3 = < or N1 or M1).

^¶^ cumulative-average method.

* For cancer cases diagnosed after 2004.

Inverse associations between a higher AIDI score and RCC risk were suggested both for localised and advanced RCC, but the estimates were not statistically significant. If anything, a slightly more convincing pattern was observed in localised RCC in the repeated measure analysis with statistically significant trends.

### Women

The analysis revealed a statistically significant trend showing that higher adherence to an anti-inflammatory diet was associated with a reduced risk of renal cell carcinoma (RCC) among women, with the most substantial protective effect observed in the highest AIDI quartile (HR: 0.47, 95% CI: 0.30, 0.75) in the fully adjusted model (Table 2b). Although the hazard ratios across quartiles were not monotonically decreasing,the overall trend was statistically significant (*p* < 0.05). (conversely = contrarywise so confusing here) For advanced RCC, a significant decreasing trend in risk was also observed with increasing AIDI scores. The most substantial reduction was seen in the highest AIDI quartile (HR: 0.26, 95% CI: 0.11, 0.63), with a p-value for trend at 0.02. This association persisted when utilising both the 1997 and the 2009 FFQ surveys. Across all models, the results consistently indicate that a higher AIDI score, reflecting a long-term adherence to an anti-inflammatory diet, is associated with a reduced risk of RCC among women. (Table 2b).

### Men

Among men overall, inverse associations between higher AIDI scores and the risk of RCC were observed although not statistically significant and without any clear trend of lower risks with higher AIDI scores. Largely similar patterns were observed both for localised and advanced RCC (Table 2c)

### Additional analyses

In a secondary analysis, the AIDI was segmented into pro and anti-inflammatory items. In mutually adjusted analyses (anti-inflammatory components of AIDI adjusted for pro-inflammatory components and vice versa) higher compared to lower AIDI-scores rendered HR estimates similar to main analyses, but with 95% CI encompassing 1 among both men and women population (Tables 3a, b, 4a & b).

**Table 3:** a: Associations between only anti-inflammatory components of AIDI and risk for renal cell carcinoma (RCC), overall and by disease stage. HRs (95% CIs) of RCC by quartile of AIDI in 35,267, Swedish women, 1997–2020. b: Associations between only anti-inflammatory components of AIDI and risk for renal cell carcinoma (RCC), overall and by disease stage. HRs (95% CIs) of RCC by quartile of AIDI in 36,154, Swedish men, 1997–2020.

Anti Inflammatory AIDI score	Cases	Person-years	Baseline exposure-1997	Repeated measure of AIDI (1997 & 2009)¶
			Hazard ratios (95% confidence intervals)	Hazard ratios (95% confidence intervals)
			Model 3^a^	Model 3^a^
a				
RCC	148			
Q1 (0–3)	40	160,337	Reference	Reference
Q2 (3)	48	173,805	1.15 (0.75, 1.75)	0.63 (0.34, 1.17)
Q3 (4)	33	160,266	0.88 (0.55, 1.42)	0.76 (0.50, 1.14)
Q4 (5–10)	27	184,548	0.65 (0.39, 1.08)	0.57 (0.36, 0.91)
P value for trend			0.13	0.07
Localised RCC^b *^				
Q1 (0–3)	9	160,159	Reference	Reference
Q2 (3)	12	173,552	1.12 (0.62, 2.00)	0.50 (0.11, 2.18)
Q3 (4)	9	160,058	0.80 (0.41, 1.55)	1.20 (0.56, 2.57)
Q4 (5-10)	13	184,434	0.79 (0.41, 1.52)	1.27 (0.56, 2.87)
P value for trend			0.59	0.65
Advanced RCC^c *^				
Q1 (0–3)	16	160,202	Reference	Reference
Q2 (3)	21	173,639	1.10 (0.68, 1.79)	1.06 (0.50, 2.26)
Q3 (4)	16	160,138	0.83 (0.48, 1.41)	0.65 (0.34, 1.25)
Q4 (5–10)	9	184,318	0.43 (0.23, 0.84)	0.32 (0.14, 0.77)
*P* value for trend			0.03	0.05

b				
Anti Inflammatory AIDI score	Cases	Person-years	Baseline exposure-1997	Repeated measure of AIDI (1997 & 2009)¶
			Hazard ratios (95% confidence intervals)	Hazard ratios (95% confidence intervals)
			Model 3^a^	Model 3^a^
RCC	283			
Q1 (0–3)	77	146,492	Reference	Reference
Q2 (3)	75	184,187	0.77 (0.56, 1.07)	0.61 (0.38, 0.99)
Q3 (4)	74	165,458	0.86 (0.62, 1.18)	0.81 (0.60, 1.09)
Q4 (5-10)	57	162,429	0.68 (0.47, 0.99)	0.77 (0.56, 1.08)
*P* value for trend			0.20	0.14
Localised RCC^b*^				
Q1 (0–3)	31	146,204	Reference	Reference
Q2 (3)	26	183,912	0.82 (0.56, 1.20)	0.94 (0.51, 1.75)
Q3 (4)	29	165,179	0.91 (0.62, 1.33)	0.65 (0.39, 1.10)
Q4 (5-10)	19	162,114	0.57 (0.36, 0.91)	0.71 (0.41, 1.21)
P value for trend			0.11	0.33
Advanced RCC^c*^				
Q1 (0–3)	22	146,028	Reference	Reference
Q2 (3)	19	183,784	0.86 (0.57, 1.29)	0.32 (0.10, 1.06)
Q3 (4)	19	165,013	0.88 (0.58, 1.34)	0.91 (0.53, 1.57)
Q4 (5–10)	24	162,148	0.77 (4.84, 1.23)	1.07 (0.61, 1.87)
*P* value for trend			0.74	0.27

^a^Model3 adjusting for age, smoking (pack years), *BMI* education (primary, secondary, university), employment status (full time, part time, unemployed, studying, disability pension, retired), diabetes (Yes, No), hypertension (Yes, No), hypercholesterolaemia (Yes, No) chronic kidney disease status (Yes, No) and average caloric intake (centred at the mean by sex), and adjusted for *pro*-Inflammatory components AIDI score.

^b^Total RCC excluding advanced RCC (T3 = < or N1 or M1).

^c^advanced RCC (T3 = < or N1 or M1).

^¶^cumulative-average method.

*For cancer cases diagnosed after 2004.

**Table 4:** a: Associations between decreasing *pro*-inflammatory components of AIDI and risk for renal cell carcinoma (RCC), overall and by disease stage. HRs (95% CIs) of RCC by quartile of AIDI in 35,267, Swedish women, 1997–2020. b: Associations between *pro*-inflammatory components of AIDI and risk for renal cell carcinoma (RCC), overall and by disease stage. HRs (95% CIs) of RCC by quartile of AIDI in 36,154, Swedish men, 1997–2020.

a				
Pro Inflammatory AIDI score	Cases	Person-years	Baseline exposure-1997	Repeated measure of AIDI (1997 & 2009)
			Model 3^a^	Model 3^a^
RCC				
Q1 (0–2)	70	276,732	Reference	Reference
Q2 (3)	39	207,332	0.74 (0.50, 1.10)	0.81 (0.46, 1.41)
Q3 (3,5)	39	194,891	0.79 (0.52, 1.22)	0.86 (0.58, 1.28)
			- - -	0.79 (0.48, 1.30)
*P* value for trend			0.30	0.77
Localised RCC^b*^	43	276,357		
Q1 (0–2)	24	207,105	Reference	Reference
Q2 (3)	6	194,741	0.68 (0.40, 1.17)	0.88 (0.35, 2.22)
Q3 (3,5)	13		0.95 (0.55, 1.65)	0.38 (0.16, 0.93)
			- - -	1.01 (0.46, 2.23)
*P* value for trend			0.35	0.16
Advanced RCC^c*^	62			
Q1 (0-2)	31	276,358	Reference	Reference
Q2 (3)	19	207,242	0.94 (0.60, 1.48)	0.90 (0.40, 2.02)
Q3 (3,5)	12	194,697	0.77 (0.46, 1.29)	1.11 (0.62, 1.99)
			- - -	0.43 (0.17, 1.09)
*P* value for trend			0.60	0.22

b				
Pro Inflammatory AIDI score	Cases	Person-years	Baseline exposure-1997	Repeated measure of AIDI (1997 & 2009)
			Model 3^a^	Model 3^a^
RCC	283			
Q1 (0–2)	153	358,019	Reference	Reference
Q2 (3)	85	184,547	1.08 (0.82, 1.42)	1.06 (0.74, 1.52)
Q3 (3,5)	45	116,000	0.89 (0.61, 1.28)	1.07 (0.80, 1.42)
			- - -	0.84 (0.54, 1.29)
P value for trend			0.57	0.72
Localised RCC^b*^	105			
Q1 (0–2)	57	357,316	Reference	Reference
Q2 (3)	38	184,300	1.25 (0.91, 1.71)	1.36 (0.80, 2.31)
Q3 (3,5)	10	115,792	0.84 (0.54, 1.31)	1.06 (0.66, 1.69)
			- - -	0.60 (0.26, 1.36)
*P* value for trend			0.16	0.31
Advanced RCC^c*^	84			
Q1 (0–2)	51	357,179	Reference	Reference
Q2 (3)	18	183,949	0.94 (0.66, 1.36)	0.83 (0.41, 1.66)
Q3 (3,5)	15	115,845	1.06 (0.70, 1.62)	0.84 (0.49, 1.45)
			- - -	1.10 (0.55, 2.20)
*P* value for trend			0.87	0.84

^a^Model3 adjusting for age, smoking (pack year), BMI, education (primary, secondary, university), employment status (full time, part time, unemployed, studying, disability pension, retired), diabetes (Yes, No), hypertension (Yes, No), hypercholesterolaemia (Yes, No) chronic kidney disease status (Yes, No) average caloric intake (centred at the mean by sex), and adjusted for *anti*-inflammatory components of AIDI score.

^b^Total RCC excluding advanced RCC (T3 = < or N1 or M1).

^c^advanced RCC (T3 = < or N1 or M1).

^¶^cumulative-average method.

*For cancer cases diagnosed after 2004.

We further tried additionally adjusting the main model for physical activity intensity, measured in metabolic equivalents (MET) hours per day, which yielded results consistent with the main analysis. For women in the highest quartile compared to the lowest (Q4 vs. Q1), the hazard ratio (HR) was 0.53 with a 95% confidence interval (CI) of 0.32-0.87; for men, the HR was 0.85 with a 95% CI of 0.60-1.22 (see Supplementary Tables 2a and 2b). Similarly, adjusting for NSAID use also produced comparable results: for women, the HR was 0.44 with a 95% CI of 0.27-0.72; for men, the HR was 0.85 with a 95% CI of 0.60-1.22 (see Supplementary Tables 3a and 3b). Consistent associations were observed across baseline and repeated measures models, even after excluding the first two years of follow-up (Supplementary Tables 4a and 4b). In an additional analysis among non-smokers, higher AIDI scores the pattern of inverse associations largely remained compared to those in the full population (Supplementary Tables 5a and 5b)

## Discussion

In this large cohort study, we found an *inverse* association between the anti-inflammatory diet index (AIDI) and renal cell carcinoma (RCC), suggesting a beneficial influence of intake of diet with high anti-inflammatory potential. The pattern was most clear among women and the findings remained robust after adjusting for various established risk factors of RCC.

Some previous studies have examined the relationship between the pro-inflammatory potential of diet and the risk of RCC, using the literature-derived Dietary Inflammatory Index (DII). A meta-analysis of the two case-control studies performed disclosed a pooled relative risk (RR) of 1.46 (95% CI 1.16–1.85) for the highest compared to the lowest DII score [43]. This finding aligns with our study’s observation of a statistically significant association between dietary inflammatory potential and RCC risk, particularly among women. However, the DII is a literature-derived index, whereas the AIDI used in our study is empirically derived and tailored to Scandinavian dietary habits, potentially providing greater specificity to the population under study.

An Italian case-control study, part of the meta-analysis, found an odds ratio of 1.41 (95% CI: 1.02–1.97) for the highest DII quartile compared to the lowest, with stronger associations observed in women, individuals aged over 60, and those with higher BMI [28]. Similarly, our study found significant associations primarily among women, with HR estimates for men showing similar directionality but lacking statistical significance. Unlike the DII used in the Italian study, the AIDI in our research offers a population-specific advantage, as it incorporates Scandinavian food products and eating habits, potentially enhancing its relevance and applicability.

Both fruits and vegetables were components of our AIDI score, and they were thoroughly included in the FFQ (Food Frequency Questionnaire) used for the calculation of the AIDI score. The effect of fruit and vegetables on RCC risk has been evaluated previously, sometimes with conflicting results. A study conducted earlier in the SMC observed that consumption of fruits and vegetables was linked to a possible risk-reduction of RCC (RR 0.59, 95% CI 0.26, 1.34) [18] Another study conducted on a cohort of health professions reported a protective effect of consuming fruits and vegetables on RCC risk among men (RR 0.45, 95% CI 0.25–0.81, for ≥6 servings of fruit and vegetable intake per day compared to <3 servings per day). The European Prospective Investigation into Cancer and Nutrition (EPIC) study did, however, not observe any association between fruit and vegetable consumption and the risk of RCC [19]. The study reported a short follow-up time (6.2 years) and few RCC cases (306 out of 375,851 participants). A case-cohort study from the Netherlands likewise reported no statistically significant association between vegetable or fruit consumption and RCC but also reported short follow-up time (9.3 years) and few RCC cases [44]. A limitation of these studies is that they analysed either total fruit or vegetable consumption or consumption of a narrow group of vegetables such as cruciferous or green leafy vegetables.

Pooled data from 13 prospective studies, encompassing a total of 1478 incident RCC cases (709 women and 769 men), with follow-up periods ranging from 7 to 20 years, pointed to a possible protective effect of fruit and vegetable consumption [45]. The pooled multivariate relative risk for individuals consuming ≥600 grams of fruits and vegetables per day was 0.68 (95% CI 0.54–0.87) when compared to those with an intake of <200 grams per day [45]. These estimates align with our study’s findings. The sensitivity analyses in this study, which separately assessed the pro- and anti-inflammatory elements of the AIDI, produced risk estimates in the same direction. This suggests that the association between AIDI and RCC risk might not be solely attributed to anti-inflammatory foods like fruits and vegetables.

Our study has several strengths; first, it utilised an extensive population-based prospective design, spanning a follow-up period of up to 22 years. Further, we had access to comprehensive dietary data at two discrete time points, separated by a span of 12 years. In identifying cancer cases within the cohort, we utilised the high-validity Swedish Cancer Registry and diligently excluded individuals with prior cancer diagnoses diagnosis except for non-melanoma skin cancer. Furthermore, we were able to adjust our analysis for the potential confounding by well-established risk factors for RCC, including smoking, obesity, hypertension, and diabetes. We also obtained detailed data on socio-demographic characteristics, migration history, and mortality status from high-quality nationwide registries, ensuring the accuracy and robustness of our findings.

One possible source of bias is that participants with healthy eating habits (corresponding to higher AIDI) may suffer fewer other health problems and may, therefore, be less likely to be diagnosed with RCC as a secondary finding [46]. While we cannot directly address this issue, our observations were consistent when adjusting for common comorbidities. Furthermore, while not statistically significant (possibly due to small numbers), HR estimates for advanced-stage RCC, which should be less susceptible to detection bias, had the same direction as those for overall RCC and localised disease among women. Concerns regarding detection bias due to healthcare access driven by socioeconomy are alleviated by the access to free health care in Sweden as well as additional adjustments for several socioeconomic variables.

The anti-Inflammatory Diet Index (AIDI) displayed robustness across multiple dimensions. Its validation against high-sensitive C-reactive protein (hsCRP) plasma concentration in a subcohort of women confirmed its ability to accurately measure diet related to inflammatory biomarkers [36]. Furthermore, the AIDI has consistently been shown to correlate with hsCRP levels, age, and various inflammatory risk factors [34]. It is important to note that the AIDI has not been specifically validated for the male population, particularly as associations were not statistically significant among men, while estimates generally were similar in direction. Another notable strength of our study is the selection of food groups grounded in their pro and anti-inflammatory attributes. This approach encapsulates a more realistic representation of dietary consumption within the Nordic region, deviating from a focus solely on one or two food categories. Sensitivity analyses separating the pro- and anti-inflammatory components of the AIDI yielded similar HR estimates, indicating that the association is not driven by any single component of the composite score.

The differing associations observed between sexes may be attributed to several factors. First, women may report their dietary habits more accurately or differently compared to men, leading to more precise associations between diet and health outcomes in studies utilising Food Frequency Questionnaires (FFQs). Second, the Anti-inflammatory Diet Index (AIDI) used in this study is validated for women but not for men, which could influence the observed gender-specific associations. Third, the longer follow-up period for women, averaging 19.3 years, compared with 18.2 years for men, may have contributed further to the observed differences. This extended duration enhances the detection of disease incidence and provides a more thorough evaluation of the long-term impacts of diet on health. Other possible explanations include differential distribution and impact of other RCC risk factors between sexes, as well as detection bias due to health differences between men and women. Smoking and comorbidities were reported in a larger proportion of the male study population. Poorer health in the male population may be associated with an increased risk of chance detection of a small renal tumour on radiological exams performed for other purposes, which may bias the result towards null. One of the previous studies examining inflammatory diet and RCC risk reported a stronger association among women, conforming to our observation [28], while observations of fruit and vegetable consumption and RCC pooled from several studies did not report sex differences [45].

While this study has several strengths, it is crucial to acknowledge the presence of certain limitations. Among these limitations is our reliance on self-administered food frequency questionnaires to evaluate dietary intake over two distinct time frames. Self-administered questionnaires like these inherently come with the potential for measurement inaccuracies, which, in turn, can result in both the underreporting and over reporting of particular food items. This inherent lack of precision could potentially lead to underestimating or overestimating associations. This type of misclassification should, however, be non-differential and should, therefore render a conservative result (towards the null). The prospective design precludes the risk of differential misclassification/recall bias. While exact information on timing of individual questionnaires is lacking, most participants responded within a period of a few months, alleviating concerns of seasonal differences. The AIDI is designed to capture evidence-based dietary patterns associated with inflammation, with thresholds grounded in scientific research rather than specific dietary approaches. While the cutoffs, such as limiting red meat and avoiding sodas, may seem strict, they are helpful in distinguishing pro- and anti-inflammatory diets in research. If our findings are confirmed in future studies, translation of them into public health recommendations may require some flexibility, but the scientific foundation of the AIDI ensures its reliability and utility.

A limitation of our study, as shown in Supplementary Table 1A and B, is the exclusion of participants with missing data, who were generally older, had higher smoking pack-years, and lower educational attainment. This could have diluted the observed effects and may reduce the generalisability of our findings. Furthermore, an additional constraint of this study was our lack of comprehensive information about a number of potential risk factors that, while in some cases not firmly established, have been suggested to either heighten or mitigate the risk of RCC, such as RCC family history and occupational exposure to specific chemicals such as asbestos, cadmium, and organic solvents [15]. Addressing these limitations in future research endeavours could contribute to a more comprehensive understanding of the complex landscape of RCC risk factors.

## Conclusion

Our data lend support to the hypothesis that the overall inflammatory potential of the diet may influence the risk for subsequent development of RCC.

## Supplementary information

Anti-inflammatory Diet index and risk of renal cell carcinoma

## Data Availability

This study utilised data from SMIPLER (Swedish Medical Information Platform for Epidemiological Research). Access to SMIPLER data is restricted due to legal and ethical regulations. The dataset is not publicly available but can be accessed upon reasonable request and approval from the relevant data custodians. Researchers interested in accessing SMIPLER data should contact simpler@surgsci.uu.se and follow the required application procedures. For more information on data access, please visit (https://www.simpler4health.se).
