# Supplementary material for: Anti-Inflammatory Diet Index and risk of renal cell carcinoma
Source: Br J Cancer. 2025 Apr 5;132(11):1027–39. doi: 10.1038/s41416-025-03000-w (PMC12120017; doi:10.1038/s41416-025-03000-w)
Supplement: Supplementary file 1 — Anti-inflammatory Diet index and risk of renal cell carcinoma [file 41416_2025_3000_MOESM1_ESM.docx]

**Title:** **Anti-inflammatory Diet index and risk of renal cell carcinoma**

**Authors: Tahir Taj^1, *^,** **Pernilla Sundqvist^2^,** **Alicja Wolk^3^****, Katja Fall^1,3^,** **Henrik Ugge^1,2^**

**Affiliation:**

^1^ Clinical Epidemiology and Biostatistics, School of Medical Sciences, Faculty of Medicine and Health, Örebro University.

^2^ Department of Urology, Faculty of Medicine and Health, Örebro University, Örebro, Sweden

^3^ Institute of Environmental Medicine, Karolinska Institutet, Stockholm, Sweden

^*^Correspondence and requests for materials should be addressed to T.T (email: tahir.taj@oru.se)

^Missing analysis^

| **Table 1a**. Descriptive baseline characteristics of Swedish men and women (stratified) by quartiles of the anti-inflammatory diet index (AIDI; maximum score=14) | | | | | | | | | | |
| --- | --- | --- | --- | --- | --- | --- | --- | --- | --- | --- |
|  | Male (36154) | | | |  | Male dropped (9752) | | | |  |
|  | Quartiles of AIDI | | | |  | Quartiles of AIDI | | | |  |
| Characteristics | 3 | 5 | 07 | 13 | p.over all | 3 | 5 | 07 | 13 | p.over all |
| Number of participants | 14751 | 8177 | 6608 | 6618 |  | 3799 | 2237 | 1884 | 1832 |  |
| Renal cell carcinoma | 124 | 69 | 42 | 48 |  | 16 | 12 | 11 | 9 | 0.874 |
| Age at baseline ±SD, years | 59.9 (±9.5) | 61.1 (±9.8) | 61.6 (±10.0) | 61.9 (±9.9) | <0.001 | 62.4 (±9.3) | 63.3 (±9.4) | 63.8 (±9.4) | 64.2 (±9.3) | <0.001 |
| Education |  |  |  |  | <0.001 |  |  |  |  | <0.001 |
| 1. Primary | 5539 (37.5%) | 2897 (35.4%) | 2174 (32.9%) | 1681 (25.4%) |  | 1574 (42.6%) | 923 (42%) | 671 (36.4%) | 539 (30.0%) |  |
| 1. Secondary | 7328 (49.7%) | 4021 (49.2%) | 3189 (48.3%) | 3272 (49.4%) |  | 1725 (46.6%) | 1006 (46%) | 893 (48.5%) | 847 (47.1%) |  |
| 1. University | 1884 (12.8%) | 1259 (15.4%) | 1245 (18.8%) | 1665 (25.2%) |  | 399 (10.8%) | 274 (12%) | 278 (15.1%) | 411 (22.9%) |  |
| Smoking Pack-years | 13.9 (±16.3) | 12.6 (±15.5) | 11.1 (±14.8) | 9.8 (±13.9) | <0.001 | 22.1 (±15.8) | 21.0 (±14.6) | 20.0 (±15.1) | 18.0 (±15.0) | <0.001 |
| BMI | 26.2 (±3.6) | 25.9 (±3.4) | 25.6 (±3.2) | 25.2 (±3.0) |  | 25.9 (±3.4) | 25.7 (±3.1) | 25.5 (±3.2) | 25.3 (±2.9) |  |
| 1. Under weight (<18.5) | 90 (0.6%) | 39 (0.5%) | 38 (0.6%) | 27 (0.4%) | <0.001 | 1181 (41.8%) | 749 (45.4%) | 677 (47.3%) | 697 (30.0%) | <0.001 |
| 1. Normal weight (18.5-25) | 5795 (39.3%) | 3506 (42.9%) | 3075 (46.5%) | 3313 (50.1%) |  | 304 (10.8%) | 157 (9.5%) | 106 (7.4%) | 82 (47.1%) |  |
| 1. Over weight (25-30) | 6900 (46.8%) | 3823 (46.8%) | 2948 (44.6%) | 2870 (43.4%) |  | 1327 (47.0%) | 743 (45.0%) | 644 (45.0%) | 642 (22.9%) |  |
| 1. Obese (>30) | 1966 (13.3%) | 809 (9.9%) | 547 (8.3%) | 408 (6.2%) |  | 12 (0.4%) | 1 (0.1%) | 4 (0.3%) | 4 (0.3%) |  |
| Employment |  |  |  |  | <0.001 |  |  |  |  | <0.001 |
| 1. Full time | 7609 (51.6%) | 4020 (49.2%) | 3219 (48.7%) | 3319 (50.2%) |  | 1645 (44.7%) | 918 (41.9%) | 755 (40.9%) | 718 (39.9%) |  |
| 1. Part time | 441 (3.0%) | 243 (3.0%) | 194 (2.9%) | 180 (2.7%) |  | 122 (3.3%) | 53 (2.4%) | 60 (3.3%) | 65 (3.6%) |  |
| 1. Unemployed | 862 (5.8%) | 392 (4.8%) | 254 (3.8%) | 209 (3.2%) |  | 172 (4.7%) | 80 (3.7%) | 48 (2.6%) | 46 (2.6%) |  |
| 1. Studying | 65 (0.4%) | 25 (0.3%) | 24 (0.4%) | 24 (0.4%) |  | 9 (0.2%) | 4 (0.2%) | 5 (0.3%) | 1 (0.1%) |  |
| 1. Disability pension | 925 (6.3%) | 397 (4.8%) | 271 (4.1%) | 217 (3.3%) |  | 211 (5.7%) | 109 (5.0%) | 76 (4.1%) | 65 (3.6%) |  |
| 1. Retired | 4849 (32.9%) | 3100 (37.9%) | 2646 (40.0%) | 2669 (40.3%) |  | 1522 (41.3%) | 1028 (46.9%) | 900 (48.8%) | 904 (50.3%) |  |
| Ever diagnosed Hypertension |  |  |  |  | 0.083 |  |  |  |  | 0.449 |
| 1. Yes | 920 (6.2%) | 585 (7.2%) | 447 (6.8%) | 1050 (15.9%) |  | 663 (17. 5%) | 378 (16.9%) | 320 (83.0%) | 288 (15.7%) |  |
| Ever diagnosed chronic kidney disease |  |  |  |  | 0.083 |  |  |  |  | 0.422 |
| 1. Yes | 1026 (7.0%) | 555 (6.8%) | 433 (6.6%) | 388 (5.9%) |  | 307 (%8.1) | 184 (%8.2) | 132 (7.0%) | 138 (7.5%) |  |
| Ever diagnosed Hypercholesterolemia |  |  |  |  | 0.009 |  |  |  |  | 0.627 |
| 1. Yes | 1433 (9.7%) | 851 (10.4%) | 633 (9.6%) | 730 (11.0%) |  | 2356 (9.4%) | 194 (%8.7) | 171 (9.1%) | 180 (9.8%) |  |
| Ever diagnosed with Diabetes |  |  |  |  | 0.057 |  |  |  |  | 0.394 |
| 1. Yes | 920 (6.2%) | 585 (7.2%) | 447 (6.8%) | 439 (6.6%) |  | 221 (5.8%) | 131 (5.9%) | 130 (6.9%) | 108 (5.9%) |  |
| Energy intake kcal day | 2671 (±928) | 2652 (±891) | 2657 (±928) | 2692 (±840) | 0.035 | 565 (±991) | 2590 (±916) | 2544 (±886) | 2616 (±896) | 0.089 |
| Food consumption, |  |  |  |  |  |  |  |  |  |  |
| anti-inflammatory items |  |  |  |  |  |  |  |  |  |  |
| 1. Fruits and vegetables ^a^ | 3.3 (±1.9) | 3.6 (±2.3) | 4.1 (±2.6) | 5.0 (±2.9) | 0.001 | 3.2 (±2.2) | 3.5 (±2.4) | 3.8 (±2.6) | 4.7 (±2.9) | 0.001 |
| 1. Whole grain bread ^a^ | 4.2 (±3.8) | 5.0 (±3.9) | 5.1 (±3.7) | 5.5 (±3.7) | <0.001 | 4.0 (±3.6) | 4.9 (±3.6) | 5.2 (±3.7) | 5.5 (±3.6) | <0.001 |
| 1. Breakfast cereal ^a^ | 0.5 (±0.6) | 0.7 (±0.7) | 0.8 (±0.7) | 1.1 (±0.8) | 0.001 | 0.5 (±0.6) | 0.7 (±0.6) | 0.8 (±0.7) | 1.1 (±0.7) | 0.001 |
| 1. Tea ^a^ | 0.6 (±1.1) | 0.6 (±1.2) | 0.7 (±1.4) | 0.9 (±1.6) | <0.001 | 0.6 (±1.1) | 0.6 (±1.5) | 0.6 (±1.1) | 0.8 (±1.4) | <0.001 |
| 1. Coffee ^a^ | 3.3 (±2.5) | 3.5 (±2.4) | 3.5 (±2.3) | 3.4 (±2.0) | <0.001 | 3.0 (±2.4) | 3.4 (±2.3) | 3.4 (±2.1) | 3.4 (±1.9) | <0.001 |
| 1. Chocolate ^b^ | 0.8 (±1.3) | 1.0 (±1.5) | 1.1 (±1.7) | 1.3 (±1.8) | <0.001 | 0.8 (±1.4) | 1.0 (±1.6) | 1.0 (±1.5) | 1.3 (±1.9) | <0.001 |
| 1. Nuts ^b^ | 0.2 (±0.5) | 0.3 (±0.7) | 0.3 (±1.0) | 0.5 (±1.2) | <0.001 | 0.2 (±0.6) | 0.3 (±0.7) | 0.3 (±0.6) | 0.5 (±1.4) | <0.001 |
| 1. Wine ^b^ | 1.0 (±1.6) | 1.3 (±1.8) | 1.6 (±1.9) | 2.2 (±2.1) | 0.001 | 1.0 (±1.8) | 1.3 (±1.9) | 1.5 (±1.9) | 2.0 (±2.0) | 0.001 |
| 1. Beer ^b^ | 5.1 (±7.6) | 5.6 (±7.0) | 5.7 (±6.4) | 6.0 (±5.9) | <0.001 | 5.0 (±8.1) | 5.7 (±7.4) | 5.5 (±5.9) | 5.7 (±5.4) | <0.001 |
| 1. Low fat cheese ^b^ | 0.3 (±1.2) | 0.6 (±1.5) | 0.9 (±1.8) | 1.3 (±1.9) | <0.001 | 0.5 (±1.5) | 0.8 (±1.7) | 1.2 (±2.0) | 1.6 (±2.0) | <0.001 |
| 1. Use of olive/canola oil ^b^ | 0.1 (±0.2) | 0.1 (±0.3) | 0.2 (±0.4) | 0.3 (±0.5) | 0.001 | 0.1 (±0.2) | 0.1 (±0.3) | 0.2 (±0.4) | 0.3 (±0.5) | 0.001 |
| Pro-inflammatory items |  |  |  |  |  |  |  |  |  |  |
| 1. Unprocessed meat ^a^ | 0.5 (±0.4) | 0.5 (±0.4) | 0.4 (±0.3) | 0.4 (±0.3) | <0.001 | 0.5 (±0.4) | 0.5 (±0.4) | 0.4 (±0.3) | 0.4 (±0.3) | <0.001 |
| 1. Processed meat ^a^ | 0.9 (±0.7) | 0.8 (±0.7) | 0.7 (±0.7) | 0.6 (±0.6) | 0.001 | 0.9 (±0.7) | 0.8 (±0.7) | 0.7 (±0.6) | 0.6 (±0.6) | <0.001 |
| 1. Soft drinks ^a^ | 1.4 (±1.7) | 1.1 (±1.6) | 0.9 (±1.5) | 0.7 (±1.2) | <0.001 | 1.0 (±1.5) | 0.6 (±1.3) | 0.4 (±1.0) | 0.3 (±0.7) | <0.001 |
| 1. Offal ^b^ | 0.3 (±0.6) | 0.2 (±0.6) | 0.1 (±0.5) | 0.1 (±0.4) | <0.001 | 0.3 (±0.6) | 0.1 (±0.3) | 0.1 (±0.6) | 0.1 (±0.4) | <0.001 |
| 1. Chips ^b^ | 1.8 (±1.7) | 1.6 (±1.7) | 1.5 (±1.9) | 1.3 (±1.7) | <0.001 | 1.8 (±2.1) | 1.6 (±2.0) | 1.4 (±1.7) | 1.2 (±1.9) | <0.001 |

^a^ food consumption per day
^b^ food consumption per week

Foods with anti-inflammatory potential and their serving definitions include: total fruits and vegetables (≥6 servings/day), tea (≥3 servings/day), coffee (≥2 servings/day), wholegrain bread (≥2 servings/day), breakfast cereal (≥1 serving/day), low-fat cheese (≥1 serving/day), olive and canola oil (>0 servings/day), chocolate (≥1 serving/day), nuts (≥2 servings/week), red wine (2–7 servings/week), and beer (2–14 servings/week). Foods with pro-inflammatory potential and their serving definitions are: unprocessed red meat (≤0.5 servings/day), processed red meat (≤0.5 servings/day), offal (no consumption), chips (no consumption), and soft drinks (no consumption). A score of 1 was allotted when the cut-off for each food category was met, and a score of 0 when it was not, resulting in an AIDI score ranging from 0 to 16.

| **Table 1b**. Descriptive baseline characteristics of Swedish men and women (stratified) by quartiles of the anti-inflammatory diet index (AIDI; maximum score=14) | | | | | | | | | | |
| --- | --- | --- | --- | --- | --- | --- | --- | --- | --- | --- |
|  | Female (35267) | | | |  | Female dropped (1992) | | | |  |
|  | Quartiles of AIDI | | | |  | Quartiles of AIDI | | | |  |
| Characteristics | 3 | 5 | 07 | 13 | p.over all | 3 | 5 | 07 | 13 | p.over all |
| Number of participants | 11448 | 7515 | 7049 | 9255 |  | 758 | 424 | 388 | 422 |  |
| Renal cell carcinoma | 70 | 26 | 27 | 25 |  | 5 | 1 | 2 | 1 | 0.568 |
| Age at baseline ±SD, years | 62.7 (±9.2) | 62.9 (±9.3) | 63.1 (±9.4) | 62.3 (±9.0) | <0.001 | 65.4 (±10.0) | 66.4(±10.0) | 66.3 (±10.1) | 64.7 (±9.9) | 0.04 |
| Education |  |  |  |  | <0.001 |  |  |  |  | <0.001 |
| 1. Primary | 5567 (49%) | 3326 (44%) | 2922 (42%) | 2876 (31%) |  | 352 (60.0%) | 194 (52.0%) | 164 (%46.7) | 134 (35.6%) |  |
| 1. Secondary | 4521 (40%) | 2967 (40%) | 2801 (40%) | 3719 (40%) |  | 180 (30.7%) | 142 (38.1%) | 138 (%39.3) | 156 (41.5%) |  |
| 1. University | 1360 (12%) | 1222 (16%) | 1326 (19%) | 2660 (29%) |  | 55 (9.4%) | 37 (9.9%) | 49 (%14.0) | 86 (22.9%) |  |
| Smoking Pack-years | 7.4 (±11.5) | 6.6 (±10.7) | 6.4 (±10.2) | 5.8 (±9.5) | <0.001 | 15.6 (±11.5) | 16.7 (±11.8) | 16.2 (±11.9) | 24.4 (±3.5) | 0.271 |
| BMI | 25.5 (±4.2) | 25.1 (±4.0) | 24.9 (3.8±) | 24.5 (3.6±) |  | 25.3(±4.1) | 24.5 (±3.8) | 24.8 (±3.5) | 24.5 (±3.5) | 0.006 |
| 1. Under weight (<18.5) | 194 (1.7%) | 125 (%) | 107 (1.5%) | 158 (1.7%) | <0.001 | 185 (47.4%) | 154 (58.6%) | 130 (%51.6) | 180 (59.2%) | 0.064 |
| 1. Normal weight (18.5-25) | 5606 (49.0%) | 4042 (%) | 3958 (56.1%) | 5582 (60.3%) |  | 43 (11.0%) | 22 (8.4%) | 25 (%9.9) | 17 (5.6%) |  |
| 1. Over weight (25-30) | 4029 (35.2%) | 2535 (%) | 2334 (33.1%) | 2814 (30.4%) |  | 152 (39.0%) | 80 (30.4%) | 90 (%35.7) | 101 (33.2%) |  |
| 1. Obese (>30) | 1619 (11.5%) | 813 (%) | 650 (9.2%) | 701 (7.6%) |  | 10 (2.6%) | 7 (2.7%) | 7 (%2.8) | 6 (2.0%) |  |
| Employment |  |  |  |  | <0.001 |  |  |  |  | 0.011 |
| 1. Full time | 3075 (26.9%) | 2203 (29.3%) | 2150 (30.5%) | 3274 (35.4%) |  | 114 (19.3%) | 85 (22.3%) | 77 (22.0%) | 99 (26.3%) |  |
| 1. Part time | 1957 (17.1%) | 1259 (16.8%) | 1104 (15.7%) | 1540 (16.6%) |  | 65 (11.0%) | 43 (11.3%) | 36 (10.3%) | 53 (14.1%) |  |
| 1. Unemployed | 431 (3.8%) | 247 (3.3%) | 233 (3.3%) | 251 (2.7%) |  | 35 (5.9%) | 24 (6.3%) | 14 (4.0%) | 15 (4.0%) |  |
| 1. House wife | 4416 (38.6%) | 2962 (39.4%) | 2867 (40.7%) | 3383 (36.6%) |  | 285 (48.2%) | 198 (51.8%) | 184 (52.6%) | 177 (46.9%) |  |
| 1. Disability pension | 1106 (9.7%) | 587 (7.8%) | 500 (7.1%) | 569 (6.2%) |  | 68 (11.5%) | 22 (5.8%) | 27 (7.7%) | 19 (5.0%) |  |
| 1. Retired | 463 (4.0%) | 257 (3.4%) | 195 (2.8%) | 238 (2.6%) |  | 24 (4.1%) | 10 (2.6%) | 12 (3.4%) | 14 (3.7%) |  |
| Ever diagnosed Hypertension |  |  |  |  | 0.473 |  |  |  |  | 0.736 |
| 1. Yes | 233 (2.0%) | 139 (1.9%) | 135 (1.9%) | 161 (1.7%) |  | 14 (1.9%) | 9 (2.1%) | 6 (1.6%) | 5 (1.2%) |  |
| Ever diagnosed Chronic Kidney disease |  |  |  |  | 0.473 |  |  |  |  | 0.061 |
| 1. Yes | 602 (5.3%) | 343 (4.6%) | 311 (4.4%) | 305 (3.3%) |  | 45 (5.9%) | 16 (3.8%) | 27 (6.9%) | 15 (3.5%) |  |
| Ever diagnosed Hypercholesterolemia |  |  |  |  | 0.123 |  |  |  |  | 0.613 |
| 1. Yes | 331 (2.9%) | 193 (2.6%) | 219 (3.1%) | 291 (3.1%) |  | 15 (2.0%) | 11 (2.6%) | 5 (1.3%) | 8 (1.9%) |  |
| Ever diagnosed with Diabetes |  |  |  |  | 0.004 |  |  |  |  | 0.108 |
| 1. Yes | 485 (4.2%) | 315 (4.2%) | 300 (4.3%) | 312 (3.4%) |  | 36 (4.8%) | 28 (6.6%) | 20 (5.2%) | 34 (8.1%) |  |
| Energy intake kcal day | 1686 (±570) | 1711 (±575) | 1730 (±557) | 1830 (±558) | <0.001 | 1377 (±954) | 1599 (±656) | 1617 (±585) | 1762 (±574) | <0.001 |
| Food consumption, |  |  |  |  |  |  |  |  |  |  |
| anti-inflammatory items |  |  |  |  |  |  |  |  |  |  |
| 1. Fruits and vegetables ^a^ | 4.1 (±2.3) | 4.6 (±2.7) | 5.1 (±2.9) | 6.4 (±3.3) | 0.001 | 3.2 (±2.9) | 4.3 (±3.2) | 4.7 (±3.2) | 6.0 (±3.3) | 0.001 |
| 1. Whole grain bread ^a^ | 3.3 (±3.0) | 3.8 (2±2.8) | 4.1 (±2.8) | 4.3 (±3.0) | <0.001 | 2.6 (±0.1) | 3.5 (±2.6) | 3.8 (±2.8) | 4.5 (±0.7) | <0.001 |
| 1. Breakfast cereal ^a^ | 0.6 (±0.6) | 0.7 (±0.7) | 0.8 (±0.7) | 1.0 (±0.7) | 0.001 | 0.5 (±0.6) | 0.6 (±0.7) | 0.8 (±0.8) | 1.0 (±0.7) | 0.001 |
| 1. Tea ^a^ | 0.6 (±1.0) | 0.6 (±1.2) | 0.7 (±1.3) | 0.9 (±1.6) | <0.001 | 0.4 (±0.8) | 0.5 (±1.2) | 0.6 (±1.0) | 0.8 (±1.4) | <0.001 |
| 1. Coffee ^a^ | 2.9 (±2.3) | 3.1 (±2.0) | 3.2 (±2.3) | 3.1 (±1.9) | <0.001 | 2.2 (±2.7) | 2.9 (±2.0) | 3.5 (±3.6) | 3.2 (±2.8) | <0.001 |
| 1. Chocolate ^b^ | 0.7 (±1.1) | 0.9 (±1.4) | 0.9 (±1.5) | 1.1 (±1.6) | <0.001 | 0.6 (±1.5) | 0.7 (±1.4) | 0.9 (±1.8) | 0.9 (±1.4) | <0.001 |
| 1. Nuts ^b^ | 0.2 (±05) | 0.3 (±0.6) | 0.3 (±0.6) | 0.4 (±1.1) | <0.001 | 0.2 (±0.9) | 0.2 (±0.9) | 0.3 (±1.2) | 0.4 (±1.3) | 0.005 |
| 1. Wine ^b^ | 1.0 (±1.6) | 1.3 (±1.7) | 1.5 (±1.7) | 2.1 (±1.9) | 0.001 | 0.6 (±1.1) | 1.0 (±1.4) | 1.2 (±1.9) | 1.7 (±1.7) | 0.001 |
| 1. Beer ^b^ | 1.5 (±3.6) | 1.9 (±3.5) | 2.3 (±3.7) | 2.9 (±4.0) | <0.001 | 0.9 (±2.5) | 1.3 (±2.7) | 2.1 (±6.0) | 2.9 (±4.3) | <0.001 |
| 1. Low fat cheese ^b^ | 0.4 (±1.1) | 0.8 (±1.5) | 1.0 (±1.9) | 1.4 (±1.8) | <0.001 | 0.5 (±1.3) | 1.3 (±1.6) | 1.6 (±2.2) | 2.2 (±2.0) | <0.001 |
| 1. Use of olive/canola oil ^b^ | 0.1 (±0.3) | 0.2 (±0.4) | 0.2 (±0.4) | 0.4 (±0.5) | 0.001 | 0.1 (±0.2) | 0.1 (±0.3) | 0.2 (±0.4) | 0.4 (±0.5) | 0.001 |
| Pro-inflammatory items |  |  |  |  |  |  |  |  |  |  |
| 1. Unprocessed meat ^a^ | 0.5 (±0.4) | 0.4 (±0.3) | 0.4 (±0.3) | 0.4 (±0.3) | <0.001 | 0.5 (±0.6) | 0.4 (±0.5) | 0.4 (±0.3) | 0.3 (±0.3) | <0.001 |
| 1. Processed meat ^a^ | 0.8 (±0.6) | 0.7 (±0.6) | 0.6 (±0.6) | 0.5 (±0.5) | 0.001 | 0.7 (±0.7) | 0.6 (±0.7) | 0.5 (±0.5) | 0.4 (±0.5) | <0.001 |
| 1. Soft drinks ^a^ | 1.0 (±1.5) | 0.8 (±1.5) | 0.6 (±1.1) | 0.4 (±1.0) | <0.001 | 0.6 (±1.3) | 0.5 (±1.0) | 0.2 (±0.7) | 0.2 (±0.6) | <0.001 |
| 1. Offal ^b^ | 0.3 (±0.6) | 0.2 (±0.6) | 0.1 (±0.4) | 0.1 (±0.4) | <0.001 | 0.3 (±1.5) | 0.2 (±0.9) | 0.1 (±0.2) | 0.1 (±0.4) | <0.001 |
| 1. Chips ^b^ | 1.5 (±1.6) | 1.3 (±1.7) | 1.1 (±1.5) | 0.9 (±1.4) | <0.001 | 1.3 (±2.4) | 1.4 (±2.4) | 0.9 (±1.4) | 1.0 (±1.8) | <0.001 |

^a^ food consumption per day
^b^ food consumption per week

Foods with anti-inflammatory potential and their serving definitions include: total fruits and vegetables (≥6 servings/day), tea (≥3 servings/day), coffee (≥2 servings/day), wholegrain bread (≥2 servings/day), breakfast cereal (≥1 serving/day), low-fat cheese (≥1 serving/day), olive and canola oil (>0 servings/day), chocolate (≥1 serving/day), nuts (≥2 servings/week), red wine (2–7 servings/week), and beer (2–14 servings/week). Foods with pro-inflammatory potential and their serving definitions are: unprocessed red meat (≤0.5 servings/day), processed red meat (≤0.5 servings/day), offal (no consumption), chips (no consumption), and soft drinks (no consumption). A score of 1 was allotted when the cut-off for each food category was met, and a score of 0 when it was not, resulting in an AIDI score ranging from 0 to 16.

# Supplementary Table 2a: Associations between anti-inflammatory diet index (AIDI) and risk for Renal cell carcinoma (RCC), overall and by stage. HRs (95% CIs) of RCC by quintiles of AIDI in 27584 Swedish women, 1997–2020 (additionally adjusted for physical activity)

|  |  |  | Hazard ratios (95% confidence intervals |  | Hazard ratios (95% confidence intervals |
| --- | --- | --- | --- | --- | --- |
| AIDI score | Cases | Person-years | Baseline exposure-1997 |  | Baseline exposure-1997 |
|  |  |  | Model 3 ^a^ |  | Model 3 ^b^ |
| RCC |  |  |  |  |  |
| Q1 (0-5) | 56 | 171254 | Reference |  | Reference |
| Q2 (6) | 19 | 112714 | 0.55 (0.33, 0.92) |  | 0.55 (0.33, 0.92) |
| Q3 (7) | 21 | 106625 | 0.66 (0.40, 1.09) |  | 0.66 (0.40, 1.09) |
| Q4 (8-14) | 23 | 150811 | 0.53 (0.32, 0.87) |  | 0.53 (0.32, 0.87) |
| P value for trend |  |  | 0.02 |  | 0.03 |
|  |  |  |  |  |  |
| Localized RCC *^c *^* |  |  |  |  |  |
| Q1 (0-5) | 18 | 170947 | Reference |  | Reference |
| Q2 (6) | 6 | 112604 | 0.55 (0.22, 1.38) |  | 0.55 (0.22, 1.38) |
| Q3 (7) | 3 | 106475 | 0.31 (0.09, 1.05) |  | 0.31 (0.09, 1.05) |
| Q4 (8-14) | 11 | 150738 | 0.81 (0.37, 1.76) |  | 0.81 (0.37, 1.77) |
| P value for trend |  |  | 0.22 |  | 0.22 |
|  |  |  |  |  |  |
| Advanced RCC *^d *^* |  |  |  |  |  |
| Q1 (0-5) | 25 | 170978 | Reference |  | Reference |
| Q2 (6) | 9 | 112609 | 0.58 (0.27, 1.24) |  | 0.58 (0.27, 1.25) |
| Q3 (7 | 12 | 106589 | 0.83 (0.41, 1.66) |  | 0.83 (0.41, 1.66) |
| Q4 (8-14) | 5 | 150642 | 0.28 (0.09, 0.66) |  | 0.25 (0.09, 0.66) |
| P value for trend |  |  | 0.04 |  | 0.04 |

^a^ Model3 adjusting for age, smoking (pack years), BMI (under-weight (<18.5), normal weight (18.5-25), over weight (25-30), obese (>30)), education (primary, secondary, university), employment status (full time, part time, unemployed, studying, disability pension, retired), diabetes (Yes, No), hypertension (Yes, No), hypercholesterolemia (Yes, No) and chronic kidney disease status (Yes, No) and average caloric intake ( centered at the mean by sex).
^b^ Model3 additionally adjusted for physical activity intensity, measured in metabolic equivalents (MET) hours per day

^c^ Total RCC excluding advanced RCC (T3=< or N1 or M1)
^d^ advanced RCC (T3=< or N1 or M1)
^¶^ cumulative-average method
* For cancer cases diagnosed after 2004

# Supplementary Table 2b: Associations between anti-inflammatory diet index (AIDI) and risk for Renal cell carcinoma (RCC), overall and by stage. HRs (95% CIs) of RCC by quintiles of AIDI in 27921 Swedish men, 1997–2020 (additionally adjusted for physical activity)

|  |  |  | Hazard ratios (95% confidence intervals |  | Hazard ratios (95% confidence intervals |
| --- | --- | --- | --- | --- | --- |
| AIDI score | Cases | Person-years | Baseline exposure-1997 |  | Baseline exposure-1997 |
|  |  |  | Model 3 ^a^ |  | Model 3 ^b^ |
| RCC |  |  |  |  |  |
| Q1 (0-5) | 105 | 209529 | Reference |  | Reference |
| Q2 (6) | 54 | 114710 | 0.97 (0.70, 1.34) |  | 0.97 (0.70, 1.35) |
| Q3 (7) | 34 | 94461 | 0.75 (0.51, 1.11) |  | 0.75 (0.51, 1.11) |
| Q4 (8-14) | 32 | 98613 | 0.69 (0.46, 1.04) |  | 0.69 (0.46, 1.04) |
| P value for trend |  |  | 0.21 |  | 0.21 |
|  |  |  |  |  |  |
| Localized RCC *^c *^* |  |  |  |  |  |
| Q1 (0-5) | 41 | 209078 | Reference |  | Reference |
| Q2 (6) | 16 | 114547 | 0.75 (0.42, 1.34) |  | 0.75 (0.42, 1.34) |
| Q3 (7) | 15 | 94346 | 0.89 (0.49, 1.61) |  | 0.89 (0.49, 1.61) |
| Q4 (8-14) | 8 | 98389 | 0.48 (0.22, 1.03) |  | 0.48 (0.22, 1.03) |
| P value for trend |  |  | 0.27 |  | 0.27 |
|  |  |  |  |  |  |
| Advanced RCC *^d *^* |  |  |  |  |  |
| Q1 (0-5) | 33 | 208931 | Reference |  | Reference |
| Q2 (6) | 12 | 114446 | 0.69 (0.36, 1.35) |  | 0.69 (0.36, 1.35) |
| Q3 (7 | 9 | 94235 | 0.64 (0.31, 1.35) |  | 0.64 (0.31, 1.35) |
| Q4 (8-14) | 15 | 98492 | 1.04 (0.56, 1.95) |  | 1.04 (0.56, 1.95) |
| P value for trend |  |  | 0.47 |  | 0.47 |

^a^ Model3 adjusting for age, smoking (pack years), BMI (under-weight (<18.5), normal weight (18.5-25), over weight (25-30), obese (>30)), education (primary, secondary, university), employment status (full time, part time, unemployed, studying, disability pension, retired), diabetes (Yes, No), hypertension (Yes, No), hypercholesterolemia (Yes, No) and chronic kidney disease status (Yes, No) and average caloric intake ( centered at the mean by sex)
^b^  Model3 additionally adjusted for physical activity intensity, measured in metabolic equivalents (MET) hours per day

^c^ Total RCC excluding advanced RCC (T3=< or N1 or M1)
^d^ advanced RCC (T3=< or N1 or M1)
^¶^ cumulative-average method
* For cancer cases diagnosed after 2004

# Supplementary Table 3a: Associations between anti-inflammatory diet index (AIDI) and risk for Renal cell carcinoma (RCC), overall and by stage. HRs (95% CIs) of RCC by quintiles of AIDI in 30609, Swedish women, 1997–2020 (additionally adjusted for NSAID)

|  |  |  | Hazard ratios (95% confidence intervals |  | Hazard ratios (95% confidence intervals |
| --- | --- | --- | --- | --- | --- |
| AIDI score | Cases | Person-years | Baseline exposure-1997 |  | Baseline exposure-1997 |
|  |  |  | Model 3 ^a^ |  | Model 3 ^b^ |
| RCC |  |  |  |  |  |
| Q1 (0-5) | 67 | 189638 | Reference |  | Reference |
| Q2 (6) | 23 | 125412 | 0.55 (0.34, 0.88) |  | 0.55 (0.34, 0.88) |
| Q3 (7) | 25 | 116831 | 0.66 (0.42, 1.05) |  | 0.66 (0.42, 1.05) |
| Q4 (8-14) | 22 | 162014 | 0.44 (0.27, 0.72) |  | 0.44 (0.27, 0.72) |
| P value for trend |  |  | 0.00 |  | 0.00 |
|  |  |  |  |  |  |
| Localized RCC *^c *^* |  |  |  |  |  |
| Q1 (0-5) | 21 | 189285 | Reference |  | Reference |
| Q2 (6) | 5 | 125278 | 0.40 (0.15, 1.06) |  | 0.40 (0.15, 1.06) |
| Q3 (7) | 2 | 116654 | 0.18 (0.04, 0.77) |  | 0.18 (0.04, 0.77) |
| Q4 (8-14) | 10 | 161952 | 0.68 (0.31, 1.48) |  | 0.68 (0.31, 1.48) |
| P value for trend |  |  | 0.05 |  | 0.05 |
|  |  |  |  |  |  |
| Advanced RCC *^d *^* |  |  |  |  |  |
| Q1 (0-5) | 29 | 189324 | Reference |  | Reference |
| Q2 (6) | 12 | 125324 | 0.65 (0.33, 1.28) |  | 0.65 (0.33, 1.28) |
| Q3 (7) | 14 | 116803 | 0.83 (0.43, 1.58) |  | 0.83 (0.43, 1.58) |
| Q4 (8-14) | 5 | 161850 | 0.22 (0.08, 0.57) |  | 0.22 (0.08, 0.57) |
| P value for trend |  |  | 0.02 |  | 0.02 |

^a^ Model3 adjusting for age, smoking (pack years), BMI (under-weight (<18.5), normal weight (18.5-25), over weight (25-30), obese (>30)), education (primary, secondary, university), employment status (full time, part time, unemployed, studying, disability pension, retired), diabetes (Yes, No), hypertension (Yes, No), hypercholesterolemia (Yes, No) and chronic kidney disease status (Yes, No) and average caloric intake (centered at the mean by sex)
^b^ Model3 additionally adjusted for NSAID use (Yes, No)
^c^ Total RCC excluding advanced RCC (T3=< or N1 or M1)
^d^ advanced RCC (T3=< or N1 or M1)
^¶^ cumulative-average method
* For cancer cases diagnosed after 2004

# Supplementary Table 3b: Associations between anti-inflammatory diet index (AIDI) and risk for Renal cell carcinoma (RCC), overall and by stage. HRs (95% CIs) of RCC by quintiles of AIDI in 33095, Swedish men, 1997–2020 (additionally adjusted for NSAID)

|  |  |  | Hazard ratios (95% confidence intervals |  | Hazard ratios (95% confidence intervals |
| --- | --- | --- | --- | --- | --- |
| AIDI score | Cases | Person-years | Baseline exposure-1997 |  | Baseline exposure-1997 |
|  |  |  | Model 3 ^a^ |  | Model 3 ^b^ |
| RCC |  |  |  |  |  |
| Q1 (0-5) | 117 | 248981 | Reference |  | Reference |
| Q2 (6) | 66 | 135682 | 1.05 (0.77, 1.41) |  | 1.05 (0.77, 1.41) |
| Q3 (7) | 39 | 111670 | 0.76 (0.52, 1.09) |  | 0.76 (0.52, 1.09) |
| Q4 (8-14) | 44 | 113102 | 0.85 (0.60, 1.22) |  | 0.85 (0.60, 1.22) |
| P value for trend |  |  | 0.35 |  | 0.35 |
|  |  |  |  |  |  |
| Localized RCC *^c *^* |  |  |  |  |  |
| Q1 (0-5) | 46 | 248454 | Reference |  | Reference |
| Q2 (6) | 22 | 135520 | 0.91 (0.55, 1.51) |  | 0.91 (0.55, 1.51) |
| Q3 (7) | 17 | 111546 | 0.86 (0.49, 1.51) |  | 0.86 (0.49, 1.51) |
| Q4 (8-14) | 14 | 112824 | 0.73 (0.39, 1.34) |  | 0.73 (0.39, 1.34) |
| P value for trend |  |  | 0.77 |  | 0.77 |
|  |  |  |  |  |  |
| Advanced RCC *^d *^* |  |  |  |  |  |
| Q1 (0-5) | 39 | 248312 | Reference |  | Reference |
| Q2 (6) | 12 | 135321 | 0.58 (0.30, 1.11) |  | 0.58 (0.30, 1.11) |
| Q3 (7) | 10 | 111404 | 0.59 (0.30, 1.20) |  | 0.60 (0.30, 1.20) |
| Q4 (8-14) | 19 | 112883 | 1.13 (0.64, 1.99) |  | 1.14 (0.65, 2.00) |
| P value for trend |  |  | 0.14 |  | 0.14 |

^a^ Model3 adjusting for age, smoking (pack years), BMI (under-weight (<18.5), normal weight (18.5-25), over weight (25-30), obese (>30)), education (primary, secondary, university), employment status (full time, part time, unemployed, studying, disability pension, retired), diabetes (Yes, No), hypertension (Yes, No), hypercholesterolemia (Yes, No) chronic kidney disease status (Yes, No) and average caloric intake ( centered at the mean by sex)
^b^ Model3 additionally adjusted for NSAID use (Yes, No)
^c^ Total RCC excluding advanced RCC (T3=< or N1 or M1)
^d^ advanced RCC (T3=< or N1 or M1)
^¶^ cumulative-average method
* For cancer cases diagnosed after 2004

# Supplementary Table 4a: Associations between anti-inflammatory diet index (AIDI) and risk for Renal cell carcinoma (RCC), overall and by stage. HRs (95% CIs) of RCC by quintiles of AIDI in 35267, Swedish women, 1997–2020 (first two year of followup dropped )

|  |  |  | Baseline exposure-1997 |  | Repeated measure of AIDI (1997 & 2009)^¶^ |
| --- | --- | --- | --- | --- | --- |
|  |  |  | Hazard ratios (95% confidence intervals) |  | Hazard ratios (95% confidence intervals) |
|  | Cases | Person-years | Model 3 ^c^ |  | Model 3 ^c^ |
| RCC | 136 | 678486 |  |  |  |
| Q1 (0-5) | 64 | 216437 | Reference |  | Reference |
| Q2 (6) | 23 | 143314 | 0.57 (0.35, 0.92) |  | 0.79 (0.51, 1.24) |
| Q3 (7) | 25 | 135115 | 0.68 (0.42, 1.07) |  | 0.54 (0.34, 0.87) |
| Q4 (8-14) | 24 | 183620 | 0.49 (0.31, 0.79) |  | 0.59 (0.37, 0.96) |
| P value for trend |  |  | 0.01 |  | 0.04 |
|  |  |  |  |  |  |
| Localized RCC *^d *^* | 43 | 677734 |  |  |  |
| Q1 (0-5) | 21 | 216084 | Reference |  | Reference |
| Q2 (6) | 7 | 143180 | 0.54 (0.23, 1.29) |  | 0.65 (0.29, 1.48) |
| Q3 (7) | 3 | 134930 | 0.25 (0.08, 0.86) |  | 0.33 (0.13, 0.86) |
| Q4 (8-14) | 12 | 183540 | 0.75 (0.36, 1.57) |  | 0.81 (0.37, 1.78) |
| P value for trend |  |  | 0.12 |  | 0.14 |
|  |  |  |  |  |  |
| Advanced RCC *^e *^* | 62 | 677828 |  |  |  |
| Q1 (0-5) | 29 | 216122 | Reference |  | Reference |
| Q2 (6) | 12 | 143196 | 0.65 (0.33, 1.28) |  | 1.07 (0.56, 2.05) |
| Q3 (7) | 15 | 135080 | 0.87 (0.46, 1.64) |  | 0.78 (0.40, 1.51) |
| Q4 (8-14) | 6 | 183429 | 0.26 (0.11, 0.64) |  | 0.43 (0.18, 1.01) |
| P value for trend |  |  | 0.03 |  | 0.17 |

^a^ Model1 adjusting for age
^b^ Model2 adjusting for age smoking (pack years), BMI, education, employment status and average caloric intake (centered at the mean by sex)
^c^ Model3 adjusting for age, smoking (pack years), BMI, education, employment status, diabetes, hypertension, hypercholesterolemia, chronic kidney disease status (Yes, No), and average caloric intake (centered at the mean by sex)
^d^ Total RCC excluding advanced RCC (T3=< or N1 or M1)
^e^ advanced RCC (T3=< or N1 or M1)
^¶^ cumulative-average method
* For cancer cases diagnosed after 2004

# Supplementary Table 4b: Associations between anti-inflammatory diet index (AIDI) and risk for Renal cell carcinoma (RCC), overall and by stage. HRs (95% CIs) of RCC by quintiles of AIDI in 36154, Swedish men, 1997–2020 (first two year of followup dropped )

|  |  |  | Baseline exposure-1997 | Repeated measure of AIDI (1997 & 2009)^¶^ |
| --- | --- | --- | --- | --- |
| AIDI score |  |  | Hazard ratios (95% confidence intervals) | Hazard ratios (95% confidence intervals) |
|  | Cases | Person-years | Model 3 ^c^ | Model 3 ^c^ |
| RCC | 258 | 657731 |  |  |
| Q1 (0-5) | 120 | 268395 | Reference | Reference |
| Q2 (6) | 57 | 147120 | 0.87 (0.63, 1.19) | 0.82 (0.60, 1.13) |
| Q3 (7) | 40 | 120519 | 0.76 (0.53, 1.08) | 0.65 (0.47, 0.90) |
| Q4 (8-14) | 41 | 121697 | 0.78 (0.54, 1.12) | 0.74 (0.51, 1.07) |
| P value for trend |  |  | 0.35 | 0.06 |
|  |  |  |  |  |
| Localized RCC *^d *^* | 105 | 656574 |  |  |
| Q1 (0-5) | 50 | 267867 | Reference | Reference |
| Q2 (6) | 22 | 146919 | 0.83 (0.50, 1.38) | 0.83 (0.51, 1.35) |
| Q3 (7) | 18 | 120395 | 0.85 (0.50, 1.47) | 0.52 (0.30, 0.89) |
| Q4 (8-14) | 15 | 121393 | 0.74 (0.41, 1.34) | 0.87 (0.49, 1.54) |
| P value for trend |  |  | 0.74 | 0.12 |
|  |  |  |  |  |
| Advanced RCC *^e *^* | 84 | 656139 |  |  |
| Q1 (0-5) | 39 | 267665 | Reference | Reference |
| Q2 (6) | 14 | 146758 | 0.67 (0.36, 1.23) | 0.72 (0.40, 1.31) |
| Q3 (7) | 10 | 120245 | 0.59 (0.29, 1.19) | 0.69 (0.39, 1.22) |
| Q4 (8-14) | 21 | 121470 | 1.25 (0.72, 2.15) | 1.04 (0.56, 1.91) |
| P value for trend |  |  | 0.13 | 0.43 |

^a^ Model1 adjusting for age
^b^ Model2 adjusting for age, smoking (pack years), BMI, education (primary, secondary, university), employment status (full time, part time, unemployed, studying, disability pension, retired) and average caloric intake (centered at the mean by sex)
^c^ Model3 adjusting for age, smoking (pack years), BMI, education (primary, secondary, university), employment status (full time, part time, unemployed, studying, disability pension, retired), diabetes, hypertension, hypercholesterolemia, chronic kidney disease status (Yes, No), and average caloric intake (centered at the mean by sex)
^d^ Total RCC excluding advanced RCC (T3=< or N1 or M1)
^e^ advanced RCC (T3=< or N1 or M1)
^¶^ cumulative-average method
* For cancer cases diagnosed after 2004

# Supplementary Table 5a: Associations between anti-inflammatory diet index (AIDI) and risk for Renal cell carcinoma (RCC), overall and by stage. HRs (95% CIs) of RCC by quintiles of AIDI in 35267, Swedish women, 1997–2020 (Non smoker only)

|  |  |  | Baseline exposure-1997 |  | Repeated measure of AIDI (1997 & 2009)^¶^ |
| --- | --- | --- | --- | --- | --- |
|  |  |  | Hazard ratios (95% confidence intervals) |  | Hazard ratios (95% confidence intervals) |
| AIDI score | Cases | Person-years | Model 3 ^c^ |  | Model 3 ^c^ |
| RCC | 81 | 372338 |  |  |  |
| Q1 (0-5) | 34 | 119273 | Reference |  | Reference |
| Q2 (6) | 16 | 79442 | 0.74 (0.41, 1.34) |  | 0.78 (0.43, 1.40) |
| Q3 (7) | 18 | 74529 | 0.91 (0.51, 1.61) |  | 0.69 (0.40, 1.22) |
| Q4 (8-14) | 13 | 99094 | 0.51 (0.27, 0.97) |  | 0.46 (0.23, 0.92) |
| P value for trend |  |  | 0.20 |  | 0.17 |
|  |  |  |  |  |  |
| Localized RCC *^d *^* | 19 | 371914 |  |  |  |
| Q1 (0-5) | 11 | 119101 | Reference |  | Reference |
| Q2 (6) | 5 | 79380 | 0.75 (0.26, 2.17) |  | 0.57 (0.19, 1.68) |
| Q3 (7) | 1 | 74419 | 0.17 (0.02, 1.31) |  | 0.18 (0.04, 0.83) |
| Q4 (8-14) | 2 | 99014 | 0.26 (0.06, 1.20) |  | 0.22 (0.05, 1.06) |
| P value for trend |  |  | 0.16 |  | 0.07 |
|  |  |  |  |  |  |
| Advanced RCC *^e *^* | 35 | 372037 |  |  |  |
| Q1 (0-5) | 14 | 119110 | Reference |  | Reference |
| Q2 (6) | 6 | 79364 | 0.69 (0.26, 1.79) |  | 1.02 (0.39, 2.66) |
| Q3 (7) | 9 | 74508 | 1.14 (0.49, 2.65) |  | 1.25 (0.53, 2.99) |
| Q4 (8-14) | 6 | 99055 | 0.56 (0.21, 1.49) |  | 0.57 (0.18, 1.77) |
| P value for trend |  |  | 0.50 |  | 0.53 |

^a^ Model1 adjusting for age
^b^ Model2 adjusting for age BMI, education, employment status and average caloric intake (centered at the mean by sex)
^c^ Model3 adjusting for age, smoking (pack years), BMI, education, employment status, diabetes, hypertension, hypercholesterolemia, chronic kidney disease status (Yes, No), and average caloric intake (centered at the mean by sex)
^d^ Total RCC excluding advanced RCC (T3=< or N1 or M1)
^e^ advanced RCC (T3=< or N1 or M1)
^¶^ cumulative-average method
* For cancer cases diagnosed after 2004

# Supplementary Table 5b: Associations between anti-inflammatory diet index (AIDI) and risk for Renal cell carcinoma (RCC), overall and by stage. HRs (95% CIs) of RCC by quintiles of AIDI in 36154, Swedish men, 1997–2020 (Non smoker only)

|  |  |  | Baseline exposure-1997 | Repeated measure of AIDI (1997 & 2009)^¶^ |
| --- | --- | --- | --- | --- |
| AIDI score |  |  | Hazard ratios (95% confidence intervals) | Hazard ratios (95% confidence intervals) |
|  | Cases | Person-years | Model 3 ^c^ | Model 3 ^c^ |
| RCC | 112 | 271467 |  |  |
| Q1 (0-5) | 51 | 104767 | Reference | Reference |
| Q2 (6) | 26 | 58383 | 0.93 (0.58, 1.50) | 1.04 (0.66, 1.65) |
| Q3 (7) | 18 | 52561 | 0.72 (0.42, 1.25) | 0.54 (0.31, 0.92) |
| Q4 (8-14) | 17 | 55757 | 0.66 (0.37, 1.15) | 0.71 (0.40, 1.27) |
| P value for trend |  |  | 0.41 | 0.07 |
|  |  |  |  |  |
| Localized RCC *^d *^* | 48 | 271053 |  |  |
| Q1 (0-5) | 23 | 104556 | Reference | Reference |
| Q2 (6) | 9 | 58321 | 0.75 (0.35, 1.64) | 0.64 (0.30, 1.37) |
| Q3 (7) | 10 | 52512 | 0.96 (0.45, 2.03) | 0.42 (0.18, 0.95) |
| Q4 (8-14) | 6 | 55663 | 0.58 (0.23, 1.46) | 0.89 (0.41, 1.97) |
| P value for trend |  |  | 0.65 | 0.18 |
|  |  |  |  |  |
| Advanced RCC *^e *^* | 29 | 270724 |  |  |
| Q1 (0-5) | 13 | 104420 | Reference | Reference |
| Q2 (6) | 5 | 58236 | 0.73 (0.26, 2.05) | 1.63 (0.64, 4.15) |
| Q3 (7) | 4 | 52402 | 0.69 (0.22, 2.14) | 0.78 (0.27, 2.27) |
| Q4 (8-14) | 7 | 55665 | 1.14 (0.44, 2.96) | 1.03 (0.32, 3.30) |
| P value for trend |  |  | 0.80 | 0.51 |

^c^ Model3 adjusting for age, BMI, education (primary, secondary, university), employment status (full time, part time, unemployed, studying, disability pension, retired), diabetes, hypertension, hypercholesterolemia, chronic kidney disease status (Yes, No), and average caloric intake (centered at the mean by sex)
^d^ Total RCC excluding advanced RCC (T3=< or N1 or M1)
^e^ advanced RCC (T3=< or N1 or M1)
^¶^ cumulative-average method
* For cancer cases diagnosed after 2004

# Supplement Table 6: Associations between anti-inflammatory diet index (AIDI) and risk for Renal cell carcinoma (RCC), overall and by stage. HRs (95% CIs) of RCC by quintiles of AIDI in 71,421 Swedish women & Men, 1997–2020

|  |  |  | Baseline exposure-1997 | | |  | Repeated measure of AIDI (1997 & 2009)^¶^ | | |
| --- | --- | --- | --- | --- | --- | --- | --- | --- | --- |
| AIDI score |  |  | Hazard ratios (95% confidence intervals) | | |  | Hazard ratios (95% confidence intervals) | | |
|  | Cases | Person-years | Model 1^a^ | Model 2 ^b^ | Model 3 ^c^ |  | Model 1^a^ | Model 2 ^b^ | Model 3 ^c^ |
| RCC | 431 |  |  |  |  |  |  |  |  |
| Q1 (0-5) | 194 | 485386 | Reference | Reference | Reference |  | Reference | Reference | Reference |
| Q2 (6) | 95 | 290728 | 0.81 (0.63,1.03) | 0.84 (0.66,1.07) | 0.85 (0.66,1.08) |  | 0.84 (0.66,1.07) | 0.88 (0.69,1.12) | 0.88 (0.69,1.13) |
| Q3 (7) | 69 | 255865 | 0.66 (0.50,0.87) | 0.71 (0.54,0.94) | 0.72 (0.55,0.95) |  | 0.57 (0.44,0.73) | 0.61 (0.47,0.79) | 0.62 (0.48,0.80) |
| Q4 (8-14) | 73 | 305543 | 0.59 (0.45,0.77) | 0.66 (0.51,0.87) | 0.68 (0.52,0.89) |  | 0.58 (0.44,0.77) | 0.67 (0.51,0.89) | 0.69 (0.52,0.92) |
| P value for trend |  |  | 0.00 | 0.01 | 0.02 |  | 0.00 | 0.00 | 0.00 |
|  |  |  |  |  |  |  |  |  |  |
| Localized RCC *^d *^* | 148 |  |  |  |  |  |  |  |  |
| Q1 (0-5) | 71 | 484505 | Reference | Reference | Reference |  | Reference | Reference | Reference |
| Q2 (6) | 29 | 290392 | 0.68 (0.44,1.05) | 0.72 (0.47,1.11) | 0.73 (0.47,1.12) |  | 0.71 (0.50,1.15) | 0.75 (0.49,1.14) | 0.76 (0.50,1.15) |
| Q3 (7) | 21 | 255556 | 0.56 (0.34,0.91) | 0.62 (0.38,1.00) | 0.62 (0.38,1.02) |  | 0.40 (0.28,0.71) | 0.44 (0.27,0.70) | 0.44 (0.28,0.71) |
| Q4 (8-14) | 27 | 305159 | 0.59 (0.38,0.93) | 0.70 (0.45,1.11) | 0.72 (0.46,1.14) |  | 0.64 (0.50,1.26) | 0.77 (0.49,1.22) | 0.80 (0.50,1.26) |
| P value for trend |  |  | 0.03 | 0.14 | 0.16 |  | 0.00 | 0.01 | 0.01 |
|  |  |  |  |  |  |  |  |  |  |
| Advanced RCC *^e *^* | 146 |  |  |  |  |  |  |  |  |
| Q1 (0-5) | 68 | 484341 | Reference | Reference | Reference |  | Reference | Reference | Reference |
| Q2 (6) | 26 | 290248 | 0.64 (0.41,1.00) | 0.66 (0.42,1.04) | 0.66 (0.42,1.04) |  | 0.84 (0.54,1.29) | 0.87 (0.56,1.34) | 0.87 (0.57,1.35) |
| Q3 (7) | 25 | 255556 | 0.69 (0.44,1.10) | 0.74 (0.46,1.17) | 0.75 (0.47,1.18) |  | 0.68 (0.44,1.05) | 0.73 (0.47,1.12) | 0.74 (0.48,1.14) |
| Q4 (8-14) | 27 | 305126 | 0.62 (0.40,0.97) | 0.69 (0.44,1.09) | 0.70 (0.44,1.11) |  | 0.63 (0.40,1.02) | 0.71 (0.43,1.17) | 0.73 (0.44,1.20) |
| P value for trend |  |  | 0.08 | 0.18 | 0.21 |  | 0.18 | 0.42 | 0.47 |

^a^ Model1 adjusting for age
^b^ Model2 adjusting for age smoking (pack years), BMI, education, employment status and average caloric intake (centered at the mean by sex)
^c^ Model3 adjusting for age, smoking (pack years), BMI, education, employment status, diabetes, hypertension, hypercholesterolemia, chronic kidney disease status (Yes, No), and average caloric intake (centered at the mean by sex)
^d^ Total RCC excluding advanced RCC (T3=< or N1 or M1)
^e^ advanced RCC (T3=< or N1 or M1)
^¶^ cumulative-average method
* For cancer cases diagnosed after 2004
